# Cue-Based Authenticity Appraisals in Limited-Verifiability Contexts: A Two-Study Test in Historic Automotive Events

**DOI:** 10.3390/bs16091644

**Published:** 2026-09-14

**Authors:** Chryssoula Chatzigeorgiou, Evangelos Christou, Ioanna Simeli

**Affiliations:** Department of Organization Management, Marketing and Tourism, International Hellenic University, P.O. Box 141, 57400 Thessaloniki, Greece; cchatzigeorgiou@ihu.gr (C.C.); simeli@ihu.gr (I.S.)

**Keywords:** authenticity appraisal, behavioral judgment, cue-based inference, limited verifiability, temporal transportation, nostalgic affect, sustainability legitimacy

## Abstract

Visitors often judge whether a heritage event feels genuine before they can check facts. We studied this at historic automotive events. Study 1 used 520 visitor surveys from four live European events. Study 2 was a randomized 2 × 2 brochure experiment with 640 participants. Both studies measured temporal transportation (a felt sense of being close to the era on display), nostalgic affect, and visitor-perceived authenticity appraisal. The measurement model fitted well in both studies. In Study 2, the average variance extracted for temporal transportation and nostalgic affect was marginal when considered alone. Richer sensory cueing was positively associated with temporal transportation and nostalgic affect in the field, and under random assignment richer brochure wording increased both states. Temporal transportation was positively associated with authenticity appraisal in both studies. Nostalgic affect was also related to authenticity appraisal, but more strongly when the event’s sustainability legitimacy was higher. Study 1 reports adjusted field associations. Study 2 identifies effects of the assigned descriptions. Later links among the measured states are within-session associations. The results are consistent with a bounded account. Distinct perceived cue domains were associated with past-oriented experiential presence in the field.

## 1. Introduction

Visitors to staged heritage rarely have decisive evidence at the moment they judge whether a representation feels genuine. They meet objects, materials, sensory detail, accounts of the past, and chances to take part, and they form an appraisal before provenance or historical accuracy can be checked. A spectator at a rally checkpoint, for example, sees the cars, hears the engines, and smells the fuel, but cannot verify any vehicle’s history on the spot. Consumer and tourism research similarly shows that authenticity assessments draw on indexical and iconic signs and are negotiated through staged experience (Chhabra et al., 2003; Gardiner et al., 2022; Grayson & Martinec, 2004). We use limited verifiability to describe this information environment: the visitor can look but cannot check.

Our account distinguishes two experiential states through which event cues may inform visitor-perceived authenticity appraisal; that is, the visitor’s judgment that the event’s version of the past feels genuine. Temporal transportation is a felt closeness to the represented era. Nostalgic affect is an affective relationship with the past. The two can occur together, yet they need not carry the same information. Temporal transportation refers to the experience the representation creates; nostalgia can also reflect personal memory, collective identity, or affection for a past the visitor never lived. We therefore examine separate paths from each state to authenticity appraisal. We also treat sustainability legitimacy, the perceived appropriateness of the event’s present-day sustainability stance, as a contextual boundary on the nostalgic-affect path.

The two studies provide complementary evidence, and neither identifies the state-to-appraisal paths experimentally. Study 1 estimates adjusted associations after live event exposure. Study 2 identifies randomized brochure-description contrasts and then estimates within-session associations among the post-treatment states and appraisals.

Two earlier studies frame this work. One offers a qualitative interpretation of nostalgia, authenticity, visitor motivation, and destination implications in historic car rallies (Christou et al., 2025). The other develops a broader conceptual model of Mobilized Nostalgia Tourism through historic automotive events (Christou & Simeli, 2026). This paper instead reports a quantitative two-study test of cue-based authenticity appraisal under limited verifiability. It uses new respondent-level data: a 2024 multi-event field survey and a 2025 randomized brochure experiment. The cue domains and the extension outcomes examined here correspond to design levers and outcomes proposed in that model, and the present studies test them empirically for the first time.

Historic automotive events are a demanding test bed: cue richness, public evaluation, and normative contestation are unusually visible there (Conlin & Jolliffe, 2016; Getz & Page, 2016). The logic reaches beyond this setting. Similar cue-based authenticity judgments arise in museums, recreated-history settings, historical exhibitions, and digitally staged heritage environments (Atzeni et al., 2022; Bonarou, 2021; Gardiner et al., 2022; Jin et al., 2020; Malhotra & Shin, 2025; Ou & Ho, 2024; Ross Arguedas et al., 2024; Samaniego-Chávez et al., 2025; Tang & Zhou, 2025). There too, people judge genuineness from curated cues before direct verification is practical. We ask three questions. Does richer cueing go with stronger temporal transportation and nostalgic affect? Is each state associated with visitor-perceived authenticity appraisal? And does the nostalgic-affect association vary with sustainability legitimacy? Throughout, we match the strength of each claim to the leverage of the design that supports it (Bailey et al., 2024; Blackwell et al., 2025; Rohrer, 2024).

### Behavioral Contribution

This paper makes three contributions. First, it defines visitor-perceived authenticity appraisal as a cue-based judgment formed when decisive external confirmation is not readily available. Second, it distinguishes temporal transportation from nostalgic affect and examines their separate links with authenticity appraisal in a field setting and in an experimental description setting. Third, it proposes sustainability legitimacy as a contextual qualifier of the nostalgic-affect route. The two studies test selected elements of this account in complementary ways.

## 2. Behavioral Mechanism and Hypotheses

**Figure 1:**
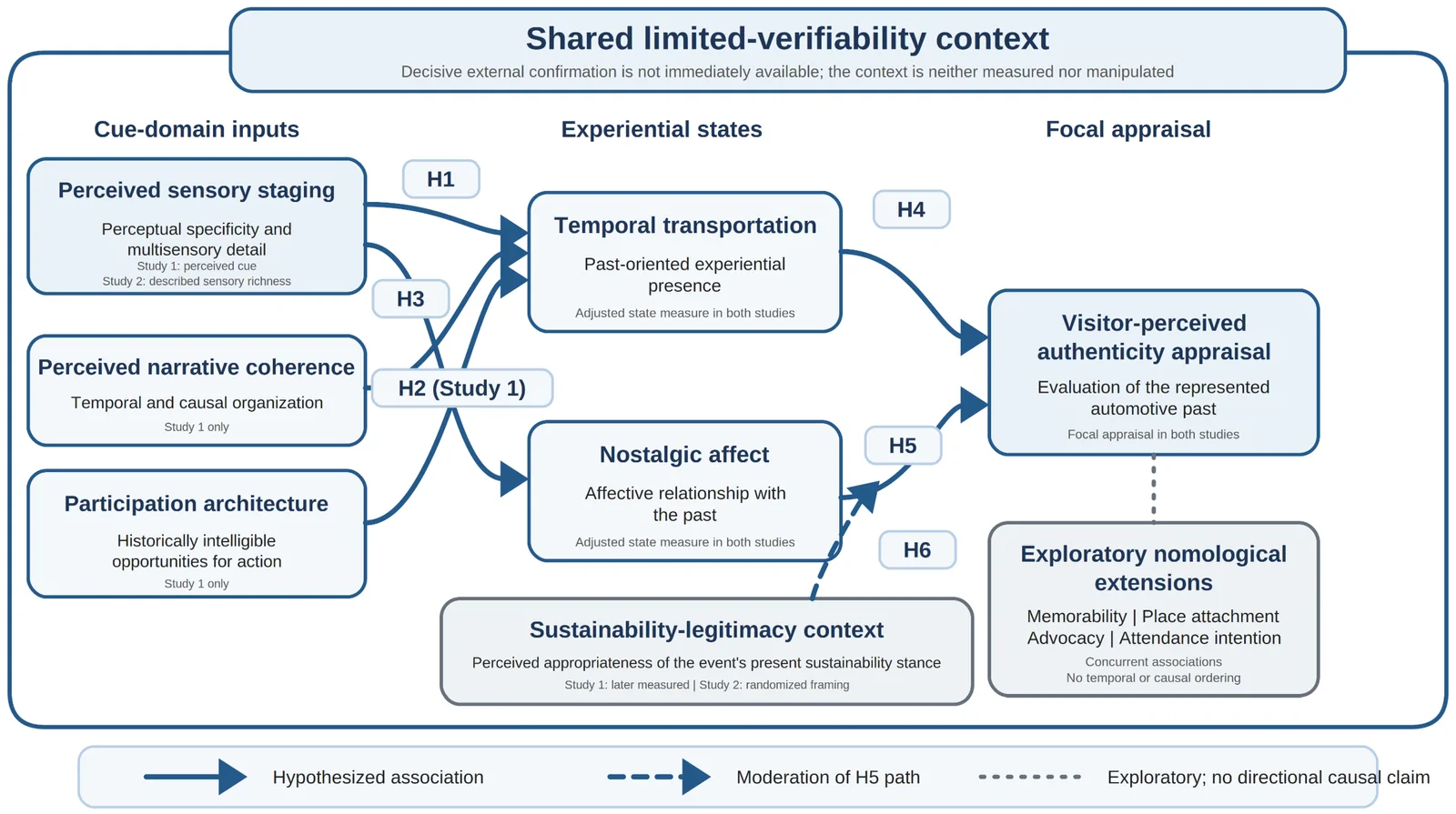
Conceptual framework. Limited verifiability denotes a shared judgment context in which decisive external confirmation is not immediately available; it was neither measured nor manipulated. Solid arrows show direct hypotheses H1–H5 (H2 covers two cue-domain paths); the dashed connector shows H6 moderation of the H5 path. Sustainability legitimacy moderates the association between nostalgic affect and authenticity appraisal (H6); constitutive main effects remain in the statistical models but are not the focal theoretical path. The dotted connector places memorability, place attachment, advocacy, and attendance intention outside the confirmatory model as exploratory nomological extensions, without directional or temporal ordering. Study 1 estimates adjusted field associations after live event exposure; Study 2 randomizes brochure descriptions of sensory richness and sustainability–legitimacy framing.

Figure 1 summarizes the proposed cue-to-state-to-appraisal model. It separates the three cue domains, treats limited verifiability as the shared judgment context, and shows sustainability legitimacy as a qualifier of the nostalgic-affect path.

### 2.1. Authenticity Appraisal in Limited-Verifiability Contexts

Authenticity research addresses several distinct questions. Object-oriented approaches ask whether an object or representation is original or demonstrably connected to its claimed source. Constructive approaches examine how signs and conventions make something recognizable as authentic. Existential approaches concern whether an experience enables a person to feel true to the self (N. Wang, 1999). Consumer research likewise distinguishes indexical signs from iconic signs (Grayson & Martinec, 2004). Indexical signs imply a factual connection with an origin; iconic signs resemble shared ideas of what an authentic offering should look or feel like. Heritage studies show that visitors evaluate staged representations through material evidence and through the experiential form in which the past is presented (Chhabra et al., 2003; Gardiner et al., 2022; Jin et al., 2020; Nath et al., 2025; Rickly & Canavan, 2024).

The dependent construct is narrower than this literature as a whole. Accordingly, the study does not adopt a single existing definition from the literature; it proposes a modified, project-specific conceptualization that synthesizes these traditions for cue-based visitor judgments in limited-verifiability contexts. Visitor-perceived authenticity appraisal is the visitor’s evaluation that the event’s representation of the automotive past feels genuine rather than merely staged, broadly true to its historical character, and credible on the cues available at the time. It is neither expert authentication of vehicles or historical claims nor a direct measure of existential authenticity. This definition follows the four appraisal items. One item uses credibility-adjacent wording, so a sensitivity analysis later checks whether the substantive pattern depends on it (AU1–AU3).

Limited verifiability specifies the setting in which the appraisal is formed. Visitors can inspect the event’s presentation but ordinarily cannot establish every vehicle’s provenance, reconstruct the historical record, or independently audit event claims while the experience is unfolding. Limited verifiability therefore describes the information environment; subjective uncertainty describes the evaluator’s confidence. The distinction matters because the theory is about how available cues inform judgment when immediate confirmation is impractical. Where decisive evidence is at hand, cue-based inference should matter less. Testing that contrast is a task for designs that vary verifiability. Limited verifiability is a shared contextual boundary, not a variable measured or manipulated in either study.

### 2.2. Distinct Cue-Domain Contributions to Temporal Transportation

We use temporal transportation as a project-specific label for past-oriented experiential presence: a momentary sense that the represented era feels near enough to be experienced from within the present setting. The construct draws on narrative transportation, generic presence, and mental time travel, but it is not interchangeable with them (Schacter et al., 2007; Stephan & Sedikides, 2024). Narrative transportation involves concentrated attention, imagery, and involvement in a story world (Green & Brock, 2000; Green & Appel, 2024). Presence research concerns the felt experience of being in an environment and able to act within it (Witmer & Singer, 1998). Temporal transportation applies these ideas to a curated past and is defined by the study items.

The three cue domains can support this state through different functions. Sensory staging supplies perceptual specificity. Sound, smell, visual detail, and material texture make the representation easier to imagine as an environment rather than as an abstract claim about the past (Shams & Seitz, 2008). Multisensory museum research links such cues with immersion and engagement (K. Guo et al., 2023; Luo et al., 2024). Narrative coherence supplies temporal and causal organization. A clear account of people, objects, places, and events helps visitors locate what they encounter within a comprehensible version of the past. Historical storytelling and interaction with guides can strengthen engagement with an authentic place (Leong et al., 2024). Participation architecture supplies behavioral affordances: things a visitor can do. Opportunities to move through a route, interact with displays, or assume a role can deepen involvement by giving visitors something historically intelligible to do. Research on museum and virtual-heritage experience similarly links self-location, possible action, interactive activity, and material presentation with presence or authenticity-related evaluation (Choi & Nam, 2024; Jiang & Fan, 2026; Jin et al., 2020; Li et al., 2024; Mitev et al., 2024; Nam et al., 2023).

These propositions concern distinct and incremental contributions. The models estimate additive relationships. Study 1 asks whether each perceived domain contributes to temporal transportation when the other domains are held constant; Study 2 tests a randomized sensory-richness description alone. Cue interactions and configurations lie outside the present analyses and remain a question for future research.

**Hypothesis** **1 (H1).** *Perceived sensory staging is positively associated with temporal transportation.*

**Hypothesis** **2 (H2).** *Perceived narrative coherence and participation architecture are positively associated with temporal transportation over and above sensory staging.*

### 2.3. Sensory Cueing and Nostalgic Affect

Nostalgia is a sentimental orientation toward the past whose emotional profile is mixed but predominantly positive (Leunissen et al., 2021; J. F. Wang, 2023; Weingarten & Wei, 2023). Its content often centers on personally meaningful people and events. Experimentally induced nostalgia can strengthen social connectedness, self-regard, and positive affect (Sedikides et al., 2008; Wildschut et al., 2006). Sensory information provides one route into this state. Music can evoke nostalgia when it is autobiographically salient (Barrett et al., 2010). Visual, auditory, olfactory, and multimodal cues retrieve autobiographical memories and shape the feeling of being brought back in time (Chu & Downes, 2000; Conway & Pleydell-Pearce, 2000; Herz, 2004; Willander et al., 2015). This makes sensory staging a plausible antecedent of nostalgic affect in a live heritage setting.

Personal recollection is only one route. Visitors can respond nostalgically to a period they did not experience directly. Vicarious nostalgia arises through imaginative engagement with a past learned from cultural representations or other people’s memories (Merchant & Rose, 2013). Collective nostalgia concerns attachment to a shared group past (Wildschut et al., 2014). Heritage-tourism research has begun to connect vicarious nostalgia with memorable experience when the represented past precedes the visitor’s lifetime (Bhogal et al., 2024). Sensory staging can therefore activate autobiographical memories for some visitors and inherited, collective, or culturally learned representations for others. H3 does not require respondents to have lived through the era being staged. These sources support plausible component routes rather than direct evidence that a staged event cue activates any one route. Their activation likely depends on personal or cultural salience. H3 therefore concerns an average relation—an adjusted association in Study 1 and an assigned-description effect in Study 2—and the studies do not test route mediation.

**Hypothesis** **3 (H3).** *Perceived sensory staging is positively associated with nostalgic affect.*

### 2.4. Experiential States as Inputs to Authenticity Appraisal

Temporal transportation and nostalgic affect may be related, yet they refer to different features of experience. Temporal transportation is defined by the representation’s capacity to create felt temporal presence. Nostalgic affect is an emotional relationship with the past and can arise from personal memory, group identity, or culturally acquired sentiment. A visitor may feel nostalgic without experiencing the event as an inhabitable historical world. Another may feel transported into an unfamiliar period without personal longing for it. Treating the constructs separately avoids reducing favorable reactions to a single heritage-experience factor.

Under limited verifiability, temporal transportation can inform authenticity appraisal because it is evidence from experience about the representation itself. When a setting supports vivid imagery, a sense of temporal closeness, and involvement in a fitting role, the setting may seem better able to sustain the represented past. Museum and virtual-heritage studies report close links among presence, object- or activity-related authenticity, and visitor evaluation (Choi & Nam, 2024; Y. Guo et al., 2024; Li et al., 2024; Nam et al., 2023). Mixed-method evidence also shows how material objects, craftsmanship, technology, emotion, and interaction contribute to experiential authenticity in heritage museums (Jin et al., 2020). These findings support a positive association between past-oriented experiential presence and authenticity appraisal.

**Hypothesis** **4 (H4).** *Temporal transportation is positively associated with visitor-perceived authenticity appraisal.*

Nostalgic affect can also contribute to authenticity appraisal. Heritage-site research links nostalgic feeling with authenticity perceptions, satisfaction, and experience sharing (Han & Bae, 2022; Muehling & Sprott, 2004; Prayag & Del Chiappa, 2023; Zhou et al., 2021). Experimental work further shows that nostalgia can heighten a person’s sense of psychological authenticity (Kelley et al., 2022). That self-focused construct differs from appraisal of an event or representation. Nostalgia can therefore supply authenticity-relevant affect without becoming equivalent to event authenticity or carrying the same evaluative weight in every context.

This distinction accords with research on feelings as information. People rely more on affect when it seems representative of the target and relevant to the judgment. They discount it when another source offers a more plausible explanation for the feeling (Greifeneder et al., 2011; Pham, 1998; Schwarz & Clore, 1983). Nostalgic affect may inform authenticity appraisal when visitors interpret it as a response to the event’s relationship with the past. This mechanism supports a positive average association while allowing the weight assigned to the feeling to vary with context.

**Hypothesis** **5 (H5).** *Nostalgic affect is positively associated with visitor-perceived authenticity appraisal.*

### 2.5. Sustainability–Legitimacy Context as a Boundary Condition

Legitimacy is an evaluative judgment that an actor, practice, or claim is appropriate within a socially constructed system of norms and expectations (Suchman, 1995). Micro-level legitimacy research treats this judgment as an active evaluation. It draws on observable signals, prevailing standards, and the interpretive logic brought to those signals (Bitektine, 2011; Tost, 2011). Experimental work shows that corporate social responsibility (CSR) and pricing signals acquire different legitimacy implications under different institutional logics (Bitektine & Song, 2023). Research on contested practices shows how conflicting normative cues prompt renewed moral evaluation (Anesa et al., 2024).

Here, sustainability legitimacy is the contextual evaluation that the event’s present-day sustainability stance appears appropriate, serious, and defensible instead of merely symbolic; it is a perceived stance, distinct from verified environmental performance and from authenticity itself. Its proposed role follows from the different information that nostalgic affect and temporal transportation carry. Historic automotive events invite affection for a mobility past that is now judged against today’s environmental expectations. Nostalgia is not uniformly aligned with sustainability: experimentally induced nostalgia can increase past orientation and reduce preference for green consumption (X. Wang & Chao, 2020). Sustainability communication can also shape perceived authenticity when audiences distinguish substantive practice from impression management (Bulmer et al., 2024; Dodd et al., 2024; Keil & Steinberger, 2024; Mair & Smith, 2021; Marco-Gardoqui et al., 2024; Mladenovic et al., 2024).

Combining legitimacy judgment with the feelings-as-information account yields the boundary prediction. A favorable sustainability–legitimacy context gives visitors fewer reasons to attribute nostalgic warmth to mere romanticization or to treat it as discordant with the event’s present conduct. Under that context, nostalgic affect should be more readily used as evidence that the event has a genuine relationship with the past. When legitimacy is weak, the same feeling may remain enjoyable while carrying less weight in the authenticity appraisal. This is a novel integrative argument. Prior research establishes the components: context-sensitive legitimacy judgments, attribution-sensitive use of affect, and possible tension between nostalgia and green preference. It has not directly demonstrated the interaction proposed here.

The moderation prediction is reserved for nostalgic affect because temporal transportation is defined as phenomenological presence in the represented era. A visitor can feel transported by a vivid or coherent representation while judging the event’s present sustainability stance unfavorably. Temporal transportation is an experience of the representation itself, and no comparable discounting mechanism is expected. Legitimacy may influence attention or engagement in other settings, so the theory leaves a temporal-transportation interaction open. It proposes only that normative context matters more for whether nostalgic feeling is accepted as evidence. The parallel TT-by-legitimacy models are exploratory specificity checks. H6 tests this predicted pathway pattern, not the attribution or diagnosticity process itself, which was not measured.

**Hypothesis** **6 (H6).** *The nostalgic-affect-authenticity-appraisal association is more positive under a more favorable sustainability–legitimacy context.*

In Study 1, sustainability legitimacy was measured after authenticity appraisal, so H6 is tested as a conditional association. In Study 2, the framing bundle is randomized, and the nostalgic-affect slope is a within-session association conditional on assignment.

### 2.6. Cross-Context Comparative Question

The literature supports separate positive paths from temporal transportation and nostalgic affect to authenticity appraisal. It does not establish a general ordering between them across heritage settings. Accordingly, we ask parallel cross-context recurrence questions:

**Research** **Question 1 (RQ1).** *Does the temporal-transportation–authenticity-appraisal association recur across the two settings, and does the nostalgic-affect–authenticity-appraisal association recur across them?*

Cross-context recurrence refers to whether the sign and substantive interpretation of each association reappear across the live-event field study and the brochure experiment, including any consistent contextual qualification. It is distinct from test–retest stability and from any claim of universal superiority.

### 2.7. Exploratory Nomological Extensions

The focal model ends with authenticity appraisal. We examine memorability, place attachment, advocacy, and attendance intention separately, to see whether the focal constructs connect plausibly with established visitor-response domains (Luong, 2024). Heritage and virtual-tourism studies link authenticity with loyalty, visit or revisit intention, and experience sharing (Atzeni et al., 2022; Choi & Nam, 2024; Han & Bae, 2022; Kolar & Zabkar, 2010). Memorability and place attachment are established dimensions of tourism and recreation experience (Kyle et al., 2005; Tung & Ritchie, 2011).

These variables are concurrent secondary correlates and receive no confirmatory hypotheses. We report them as exploratory nomological associations that connect the focal appraisal with practically relevant response domains, within the temporal and causal limits of both studies. The four extensions represent complementary nomological roles: retained experiential salience (memorability), person–place bond (place attachment), supportive recommendation or return response (advocacy), and prospective approach to the described event (attendance intention).

## 3. Materials and Methods

Study 1 is a field survey completed after live attendance at four historic automotive events. Study 2 is a randomized 2 × 2 brochure experiment that varies a sensory-vividness description and a higher/lower sustainability-legitimacy framing bundle.

In both studies, the main multi-item measures used seven-point agreement or intensity scales. Full item wording, descriptive statistics, and measurement diagnostics appear in the Supplementary Materials (Sections S1–S3). We interpret the measurement evidence from several statistics together rather than from a single cutoff (Flake & Fried, 2020; Groskurth et al., 2024). In each study, we fitted the 12 focal items to a correlated three-factor confirmatory factor analysis using covariance-based maximum likelihood (Section 3.1.4).

We computed sensitivity for the achieved samples and the specific tests they support. With N = 520 and 14 predictor terms (excluding the intercept) in the largest Study 1 model, power was 0.896 to detect a one-degree-of-freedom incremental effect of f2 = 0.02 at alpha = 0.05. With N = 640 in the 2 × 2 experiment, power was 0.966 for a one-degree-of-freedom main-effect or interaction contrast of f = 0.15 at alpha = 0.05.

### 3.1. Study 1: Field Survey of Historic Automotive Events

#### 3.1.1. Context and Procedure

We collected Study 1 data during the 2024 editions of four historic automotive events. Fieldwork covered Rallye Monte-Carlo Historique (1–4 February) at the Monaco start and at public route-control points in France, and Historic Acropolis Rally (6–9 June) in Greece. It also covered Mille Miglia (11–14 June) along the Brescia–Rome–Brescia route in Italy, and Goodwood Revival (6–8 September) at Goodwood Motor Circuit in West Sussex, United Kingdom. The settings differed in event format, route-based community contact, and destination context while sharing a focus on mobile mechanical heritage.

Trained recruiters approached visitors in busy but uncongested public areas, using a neutral invitation, and offered a QR code to the survey. Pocket-sized QR cards were the main access route, with posters at permitted information points. Recruiters could give the brief invitation in the local language, but the questionnaire and all response options were in English. Help was limited to opening the survey on the respondent’s own device; recruiters did not read or interpret items. Participation was voluntary and unpaid.

Age and event role were confirmed in the questionnaire. The survey platform did not hard-screen these fields, and ineligible records were removed during cleaning. The construct-block order was fixed. Event context and perceived cue judgments came first, followed by temporal transportation; nostalgic affect; visitor-perceived authenticity appraisal and memorability; place attachment and advocacy; sustainability legitimacy; trait nostalgia; and demographics. Items were randomized within most multi-item blocks, while the two instructed-response checks remained in fixed positions. Age and gender were optional. The cue measures record attendee perceptions rather than independently audited event characteristics. Sustainability legitimacy followed the authenticity appraisal and exploratory-extension blocks, so its Study 1 interaction is a conditional association measured later in the same session. The study was approved by the Ethics Committee of the Organizations Management, Marketing and Tourism, IHU (protocol 162/08-1-2024; 8 January 2024).

#### 3.1.2. Participants

Across the four events, 1710 attendees were approached; 715 opened the survey link or scanned the QR code, 620 consented and started, and 563 submitted complete questionnaires. Fifty-seven partial records were removed before scale construction. Among complete submissions, the documented quality rules excluded 43 records: two respondents aged under 18, seven in ineligible roles, six duplicates, ten attention-check failures, twelve straightlining or other low-quality records, and six with missing key variables. The final analytic sample, a volunteer sample, was 520, equal to 30.4% of those approached and 92.4% of complete submissions.

Respondents were aged 18–75 years (M = 44.77, SD = 12.33). The analytic event samples comprised 143 Goodwood Revival attendees, 136 Mille Miglia attendees, 130 Historic Acropolis Rally attendees, and 111 Rallye Monte-Carlo Historique attendees. The sample was 59.8% male, 37.1% female, 1.2% non-binary/other, and 1.9% preferred not to say; 66.0% were spectators and 34.0% were drivers or co-drivers. Previous attendance was reported by 42.1%. Median one-way travel distance was 458.50 km (IQR = 321.75). The analytic file contains no missing values on modeled variables; event-by-role counts and event-specific demographics appear in Supplementary Table S7 (S7_EventAnalyses sheet).

#### 3.1.3. Measures

We treated temporal transportation and nostalgic affect as state-like responses to the event. Visitor-perceived authenticity appraisal and sustainability legitimacy follow the definitions in Section 2.1 and Section 2.5. One authenticity item uses credibility-adjacent wording, so we also report a sensitivity analysis on items AU1–AU3.

*Perceived event cue judgments.* Sensory staging (four items) assessed respondents’ perceived orchestration of multisensory cues (e.g., “The event deliberately uses sound, smell, and visuals to evoke the era”). Narrative coherence (three items) captured respondents’ perceived clarity and consistency of the event’s heritage storyline. Participation architecture (3 items) captured respondents’ perceived opportunities for embodied participation and interaction along the route. Sensory-staging items were informed by sensory marketing principles (Krishna, 2012) and adapted to an event context. One item, SS4 (“Sensory details make the experience feel like stepping into the past”), is worded close to the temporal-transportation items. For that reason, a sensitivity analysis recomputes sensory staging from SS1–SS3 only (Supplementary Table S7).

*Temporal transportation (TT)*. We measured temporal transportation with a project-specific four-item scale of felt temporal nearness in a staged heritage setting. An example item is “At times during the event, I felt as if I were living in another era”. The items draw on the transportation and presence traditions and were rewritten for past-oriented experiential presence in this context. One item (TT2) is worded closest to generic absorption, so Supplementary Table S7 reports a sensitivity check that excludes it. Supplementary Section S0.3 sets out the construct boundaries.

*Nostalgic affect (NA)*. Nostalgic affect (four items) assessed the intensity of nostalgic emotion during the event (e.g., “The event made me feel a warm longing for the past”). Items were aligned with experimental nostalgia measures in social psychology (Wildschut et al., 2006) and with work on music-evoked nostalgia as a sensory trigger (Barrett et al., 2010).

Visitor-perceived authenticity appraisal was measured with four items, including “The experience felt authentic rather than staged,” and was informed by tourism-authenticity research (Chhabra et al., 2003; N. Wang, 1999). Memorability assessed the vividness and expected long-term recall of the experience, consistent with research on memorable experience and autobiographical recall (Coudounaris et al., 2025; Sutin & Robins, 2007; Tung & Ritchie, 2011). Place attachment assessed affective bonding and place identity (Kyle et al., 2005), and advocacy assessed recommendation and positive word-of-mouth intentions (Ajzen, 1991). Cronbach’s alpha for the Study 1 scales other than the three focal constructs ranged from 0.79 (narrative coherence, participation architecture, advocacy) to 0.84 (place attachment). Exact values, including memorability and the two nostalgia-proneness scales, appear in Supplementary Tables S1, S4 and S21.

In Study 1, sustainability legitimacy was measured with three items asking whether the event’s sustainability practices appeared appropriate, serious, and more than symbolic.

*Trait nostalgia proneness*. Personal and historical nostalgia proneness were measured with adapted items from established nostalgia-proneness work (Batcho, 1995, 1998; Routledge et al., 2008). These covariates help distinguish situational experience from stable trait differences.

*Controls*. We controlled for age, gender, participant role (driver/co-driver vs. spectator), repeat attendance, travel distance (log), and event fixed effects.

#### 3.1.4. Analytic Strategy

Before the substantive regressions, we evaluated the focal measurement structure for temporal transportation, nostalgic affect, and visitor-perceived authenticity appraisal with correlated three-factor confirmatory factor analyses. Study 1 also used a theory-specified seven-factor maximum-likelihood loading screen and focused confirmatory models for the perceived cue domains (Fabrigar et al., 1999). We then used unit-weighted scale means in the hypothesis models, which keeps the substantive coefficients transparent once dimensionality and within-project construct separation had been evaluated (Widaman & Revelle, 2023). The single-factor common-method screen is descriptive only and is not treated as a diagnostic test of common method bias (Podsakoff et al., 2003; Howard et al., 2024; Podsakoff et al., 2024).

We tested the hypotheses with sequenced OLS regressions and HC3 robust standard errors. Event fixed effects absorb stable mean differences across the four observed settings, but four events do not support rich event-level moderation, random-effects estimation, or cluster-robust inference (Gomes, 2022; MacKinnon et al., 2023). Leave-one-event-out and within-event estimates remain supplementary robustness checks. Bootstrap association-chain summaries describe quantities compatible with the questionnaire ordering. We do not treat them as identified mediation or temporal gatekeeping effects, because the survey was fixed-order and sustainability legitimacy was measured later (Georgeson et al., 2025; Hayes, 2018; Nguyen et al., 2022; Schuler et al., 2025).

The primary analyses were conducted in Python 3.14 using pandas, statsmodels, and scipy. The additional measurement-sensitivity analyses in Supplementary Table S22 used Python 3.12.13 and semopy 2.3.11. The code and analysis-ready materials are archived at https://doi.org/10.5281/zenodo.22214518. Within each adjusted authenticity-appraisal model, post-estimation Wald tests compare the temporal-transportation and nostalgic-affect coefficients.

### 3.2. Study 2: Brochure Experiment on Described Sensory Richness and Sustainability–Legitimacy Framing

#### 3.2.1. Design and Procedure

Study 2 was a 2 × 2 between-subjects brochure-style text-framing experiment hosted in Qualtrics and recruited through Prolific’s opt-in UK adult panel (Peer et al., 2022). Eligibility required consent, age 18–70, current UK residence, English-language participation, and one response per participant. Fieldwork ran from 14 to 17 October 2025. After the in-survey age and country checks, the Qualtrics Randomizer assigned eligible respondents to the four conditions with equal (1:1:1:1) allocation. No demographic quotas were applied; back-end monitoring continued until each cell contained 160 valid completes. The scenario described a generic, non-branded two-day historic road rally in a European destination.

Both brochure contrasts are bundled: the experiment does not isolate a single sensory component, a single legitimacy cue, or verified environmental performance. The sensory contrast changed vividness and descriptive detail as well as multisensory content. The lower-sensory text gave a general account of vehicles, checkpoints, spectators, and the town square. The higher-sensory text added engine sound, fuel and warm-oil scent, vibration, material details, and crowd ambience. The lower sustainability–legitimacy description stated that reporting was unclear, emissions and community impacts were not documented, and initiatives appeared minimal and symbolic. The higher description stated that emissions accounting was transparent and independently audited. It added cues about reduced idling and logistics impacts, heritage conservation, and community benefits.

Participants first received the brochure-imagination instruction and then read one condition-specific description. The principal post-vignette measures followed in fixed construct order: temporal transportation, nostalgic affect, visitor-perceived authenticity appraisal, and attendance intention. Six manipulation-diagnostic items came next, followed by one instructed-response attention check, required demographics, and a debrief. Required-response settings were used for eligibility, substantive items, diagnostics, the attention check, and demographics.

Random assignment identifies effects of the assigned brochure descriptions on post-vignette self-reports; the later relationships among temporal transportation, nostalgic affect, authenticity appraisal, and attendance intention are adjusted within-session associations conditional on treatment.

#### 3.2.2. Participants

Prolific invited 812 panel members; 708 opened the survey, and 688 consented, passed the in-survey eligibility checks, and were randomized, initially 172 per cell. Twenty-eight randomized respondents submitted partial questionnaires, leaving 660 complete records. Post-completion quality checks then removed five duplicate records, six attention-check failures, four records completed in under 180 s, three zero-variance straightliners, and two records flagged as bots, VPNs, or proxies. The final analytic sample contained 640 participants, 160 per cell. Participants received GBP 1.50 through Prolific for an advertised eight-minute task, equivalent to GBP 11.25 per hour; no bonus was paid.

Participants were aged 18–70 years (M = 34.90, SD = 10.28). The sample was 50.0% male, 47.0% female, 2.0% non-binary/other, and 0.9% preferred not to say. These respondents were members of an opt-in UK online panel evaluating a generic brochure description, not live event attendees or a probability sample of the UK population. Recruitment and exclusion flow, cell integrity, and demographic balance appear in Supplementary Tables S11, S12 and S14.

#### 3.2.3. Measures

Temporal transportation, nostalgic affect, and visitor-perceived authenticity appraisal used the Study 1 conceptual definitions, adapted to judgments based on the assigned brochure description. Cross-study comparison therefore spans two related elicitation modes: live attendance and brochure-based imagination. The three-item attendance-intention scale had alpha = 0.78 (Supplementary Table S6). The six manipulation-check items assessed perceived sensory richness and the perceived sustainability legitimacy of the description; they followed the principal outcomes.

### 3.3. Analytic Roadmap and Inferential Boundaries

Study 1 estimates adjusted field associations among attendee perceptions and same-session judgments. Event fixed effects adjust for mean differences across four settings but do not identify causal effects or support population-level event inference. The Study 1 sustainability–legitimacy term is more limited because the moderator was measured later in the fixed construct order (Blackwell et al., 2025).

Study 2 adds randomization at the level of the brochure description. Contrasts between the assigned sensory-vividness and sustainability–legitimacy descriptions estimate condition effects on post-vignette self-reports. Coefficients relating the post-vignette states to one another remain within-session associations conditional on treatment.

All displayed Study 2 regressions were OLS models with HC3 standard errors. Sensory richness and sustainability–legitimacy framing were coded 0/1 and their assigned-condition product was sensory × sustainability. Age was z-standardized; Female and Other gender were 0/1 indicators, with male as reference and Other combining non-binary/other with prefer-not responses. Outcomes and continuous scale predictors were standardized using the analytic-sample mean and sample SD; binary indicators were not standardized. Models A–C regressed the standardized outcome on sensory, sustainability, their product, age, Female, and Other. Model D (reduced) regressed standardized authenticity appraisal on standardized temporal transportation, sensory, sustainability, and those demographic controls. Model D (full) added standardized nostalgic affect and its product with sustainability; the ‘+ direct brochure interaction’ specification also restored sensory × sustainability.

## 4. Results

### 4.1. Measurement Structure, Reliability, and Validity

The correlated three-factor model fitted the focal items well in Study 1, chi-square(51) = 49.36, CFI = 1.000, TLI = 1.000, RMSEA = 0.000, 90% CI [0.000, 0.027], SRMR = 0.020, and in Study 2, chi-square(51) = 53.91, CFI = 0.999, TLI = 0.998, RMSEA = 0.009, 90% CI [0.000, 0.028], SRMR = 0.028. Standardized loadings ranged from 0.682 to 0.793 in Study 1 and from 0.668 to 0.777 in Study 2. The one-factor alternative was substantially worse (delta BIC = 1088.44 and 1232.60, respectively), as were all global pairwise-collapsed alternatives (minimum delta BIC = 510.32 and 602.18). Maximum HTMT was 0.428 in Study 1 and 0.284 in Study 2 (Henseler et al., 2015). Alpha and composite reliability ranged from 0.823 to 0.841 in Study 1 and from 0.785 to 0.840 in Study 2. AVE exceeded 0.50 for all Study 1 constructs and for Study 2 authenticity appraisal (Fornell & Larcker, 1981). Study 2 AVE was 0.485 for temporal transportation and 0.479 for nostalgic affect, marginally below 0.50. Taken together, reliability, loadings, fit, discriminant validity, and model comparison support the three-factor interpretation, subject to the Study 2 convergent-validity qualification just noted. For the Study 1 cue domains, the three-factor model for the ten cue items also fitted well, chi-square(32) = 37.04, CFI = 0.997, TLI = 0.996, RMSEA = 0.017, SRMR = 0.024. Composite reliability was 0.83 for sensory staging and 0.79 for narrative coherence and participation architecture, and AVE was 0.56 for each domain (Supplementary Tables S1–S3).

In Study 1, a theory-specified seven-factor maximum-likelihood loading screen of the 26 items fitted well, χ^2^(164) = 180.90, CFI = 0.997, TLI = 0.994, RMSEA = 0.014, SRMR = 0.014; oblimin rotation left all 26 primary item allocations unchanged (largest absolute secondary loading = 0.112; Supplementary Tables S2 and S3). Table 1 and Table 2 summarize measurement and correlations.

A Satorra–Bentler-scaled ML sensitivity with Huber–White sandwich standard errors was admissible in both studies (Study 1: scaled χ^2^(51) = 47.08, *p* = 0.630; Study 2: scaled χ^2^(51) = 50.08, *p* = 0.510; scaled CFI/TLI = 1.000 and RMSEA = 0.000 in both). Standardized loadings, CR, and AVE were materially unchanged across implementations (maximum absolute difference = 0.00023); standardized-loading sandwich standard errors appear in Supplementary Table S22. In 10,000 valid case bootstraps per study, every HTMT 95% upper endpoint was below 0.515. In 2000 valid case bootstraps, Study 2 AVE 90% intervals were [0.447, 0.522] for temporal transportation and [0.440, 0.519] for nostalgic affect; both crossed 0.50, so their convergent evidence remains mixed rather than being treated as a dichotomous pass/fail result (Supplementary Table S22).

### 4.2. Study 1: Cueing, Temporal Transportation, and Legitimacy Contingency

The Study 1 cue models showed positive adjusted associations with temporal transportation, which in turn was positively associated with authenticity appraisal. Sensory staging was positively associated with temporal transportation (β = 0.35, *p* < 0.001; Table 3, Model 1). The estimate held when sensory staging (SS) was recomputed from SS1–SS3 only (β = 0.34, SE = 0.04, *p* < 0.001; Supplementary Table S7). The same positive pattern appeared in leave-one-event-out and within-event re-estimations. Narrative coherence (β = 0.22, *p* < 0.001) and participation architecture (β = 0.14, *p* < 0.001) were also positive in this model. Historical nostalgia proneness was positive too (β = 0.13, *p* < 0.001), whereas personal nostalgia proneness was not (β = 0.03, *p* = 0.377).

Cue perceptions also tracked nostalgic affect through a partly distinct pattern. Sensory staging remained positive (β = 0.40, *p* < 0.001; Table 3, Model 2), narrative coherence was positive (β = 0.17, *p* < 0.001), and participation architecture was not reliable (β = 0.05, *p* = 0.191). Both personal (β = 0.13, *p* < 0.001) and historical (β = 0.15, *p* < 0.001) nostalgia proneness were positive covariates.

In the authenticity model, temporal transportation was positively associated with authenticity appraisal (β = 0.30, *p* < 0.001; Table 3, Model 3). The pattern held when TT was recomputed from TT1, TT3, and TT4 only (β = 0.29, *p* < 0.001; Supplementary Tables S7 and S15). The nostalgic-affect result depended on context. At mean perceived sustainability legitimacy, the nostalgic-affect coefficient was positive (β = 0.14, *p* < 0.001), and its interaction with perceived sustainability legitimacy was positive (β = 0.18, *p* < 0.001). The NA–authenticity association therefore strengthened as the sustainability context became more favorable.

**Figure 2:**
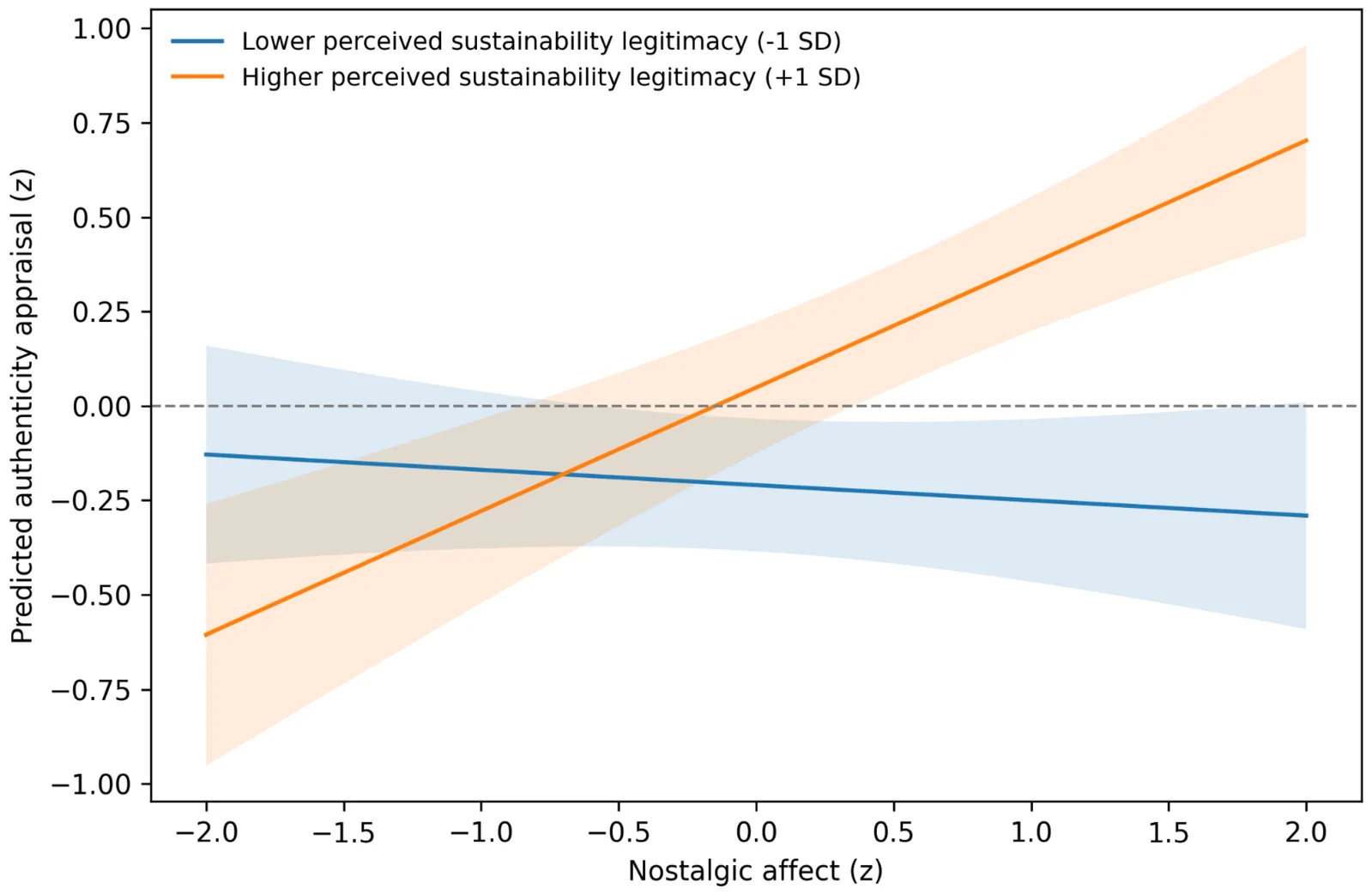
Study 1 authenticity appraisal by nostalgic affect and perceived sustainability legitimacy. Both axes are z-standardized. Lines show fitted values from the adjusted interaction model at lower (−1 standard deviation [SD]) and higher (+1 SD) perceived sustainability legitimacy, and shaded bands show 95% confidence intervals. The association between nostalgic affect and authenticity appraisal is more positive when perceived sustainability legitimacy is higher. Perceived sustainability legitimacy was measured later in the same questionnaire, so the pattern is a conditional association. Exact coefficients appear in Table S15.

Follow-up tests clarified the pattern. The nostalgic-affect simple slope was positive when perceived sustainability legitimacy was higher (+1 SD: β = 0.33, *p* < 0.001) but not when it was lower (−1 SD: β = −0.04, *p* = 0.502; Figure 2). The temporal-transportation coefficient exceeded the nostalgic-affect simple slope when legitimacy was lower (Δβ = 0.34, *p* < 0.001) or average (Δβ = 0.16, *p* = 0.016), but not when legitimacy was already high (Δβ = −0.02, *p* = 0.777; Table S15). The TT–authenticity association remained positive across leave-one-event-out and event-specific re-estimations. The NA × perceived sustainability legitimacy interaction stayed positive but was less precise in the smaller event-specific samples. No parallel TT × perceived sustainability legitimacy interaction emerged (β = −0.02, *p* = 0.654; Supplementary Table S7). Given the design, this is an absence of evidence for such an interaction rather than evidence of its absence. A direct exploratory contrast of the two interaction coefficients, reported in Supplementary Table S7 and archived at outputs/study1_authenticity_specificity_check.csv, favored the nostalgic-affect term (Δβ = 0.21, *p* = 0.003); we treat this as single-model, non-preregistered evidence rather than a confirmatory pathway test. Overall, Study 1 supports H5 at mean and higher legitimacy and gives qualified support to H6.

Study 1 exploratory nomological associations with memorability, place attachment, and advocacy were directionally consistent and are reported in Supplementary Table S19.

### 4.3. Study 2: Randomized Brochure-Condition Manipulation Checks and Contrasts

The assigned descriptions produced the intended perceived differences, and the clearest identified shifts appeared in the two experiential states, temporal transportation and nostalgic affect. The manipulation checks showed that the sensory manipulation strongly shifted perceived sensory richness while barely moving perceived sustainability legitimacy. The higher/lower sustainability–legitimacy framing bundle strongly shifted perceived sustainability legitimacy while barely moving perceived sensory richness (Supplementary Tables S9 and S10). Each manipulation moved its own target at the composite level.

High-sensory descriptions increased temporal transportation by 0.61 scale points, 95% CI [0.44, 0.78], and nostalgic affect by 0.52 points, 95% CI [0.35, 0.69] (Table 4; Supplementary Table S10). Both assigned-description effects were positive.

**Figure 3:**
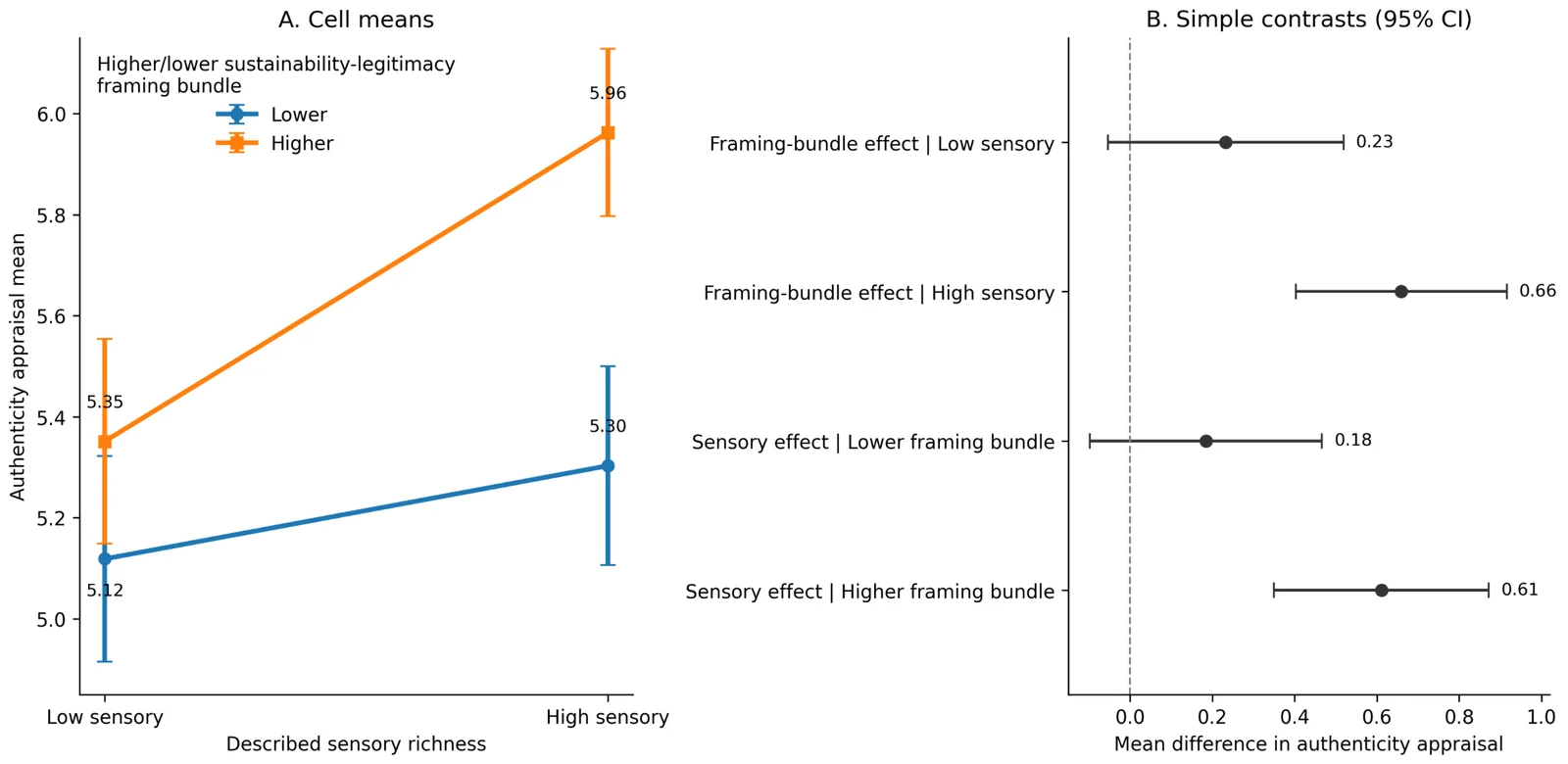
Study 2 authenticity appraisal by brochure condition. (**A**) shows unadjusted cell means with 95% confidence intervals for authenticity appraisal across the 2 × 2 brochure design. The design crossed described sensory richness with a higher/lower sustainability-legitimacy framing bundle. (**B**) shows the corresponding simple randomized brochure-condition contrasts (mean differences with 95% confidence intervals). The figure shows experimentally identified brochure-condition differences only. Table 5 (Model D) reports the adjusted within-session associations; Supplementary Table S18 reports cell means, randomized contrasts, and covariate-adjusted direct condition effects (Model C). Authenticity appraisal was highest in the high-sensory, higher-legitimacy cell (M = 5.96). The sustainability–legitimacy factor is the bundled framing described in Section 3.2.1.

The randomized authenticity pattern was narrower. It centered on the sustainability-framing contrast and on the high-sensory, higher-legitimacy cell. Authenticity appraisal was 0.45 scale points higher under the higher than the lower sustainability frame overall, 95% CI [0.25, 0.64]. It was highest in the high-sensory, higher-legitimacy cell (M = 5.96; Table 4; Figure 3). Within high sensory, the higher-framing brochure exceeded the lower-framing brochure by 0.66 points, 95% CI [0.40, 0.92]. The corresponding difference within low sensory was smaller and not independently reliable (ΔM = 0.23, 95% CI [−0.05, 0.52]). By contrast, the overall framing difference was modest for temporal transportation (ΔM = 0.23, 95% CI [0.06, 0.41]) and not independently reliable for nostalgic affect overall (ΔM = 0.09, 95% CI [−0.08, 0.26]). The lower frame contained skeptical cues rather than strong negative claims. The pattern is a relative advantage for the higher-legitimacy bundle, confined largely to the high-sensory cell.

### 4.4. Study 2: Direct Condition Model and Adjusted Within-Session Associations

Table 5 keeps Model C and Model D distinct. Model C is a covariate-adjusted randomized condition model containing the two assigned brochure indicators, their interaction, age, and the two gender indicators. In this direct condition model, the positive sensory × sustainability-framing interaction was statistically reliable (β = 0.33, *p* = 0.037). The printed sensory and sustainability coefficients are reference-category simple effects and were not independently reliable (β = 0.15, *p* = 0.186; β = 0.19, *p* = 0.099). Together with Figure 3, Model C locates the randomized authenticity advantage in the high-sensory, higher-framing brochure combination. That direct interaction is more sensitive to model specification than the TT association. When authenticity is recomputed from AU1–AU3, excluding the credibility-worded AU4 item, the Model C interaction attenuates to β = 0.28, *p* = 0.071 (Supplementary Table S7).

**Table 5:** Study 2 covariate-adjusted randomized brochure-condition models and adjusted within-session authenticity-appraisal associations conditional on treatment.

	Focal Randomized Brochure-Condition Contrast Coefficients			Adjusted Within-Session Associations Conditional on Treatment		
	Model A: TT	Model B: NA	Model C: Direct Authenticity Appraisal	Model D (Reduced): Authenticity Appraisal	Model D (Full): Authenticity Appraisal	Model D (+ Direct Brochure Interaction): Authenticity Appraisal
Described sensory richness (high = 1)	0.47 *** (0.11)	0.60 *** (0.11)	0.15 (0.11)	0.21 ** (0.08)	0.16 * (0.08)	0.03 (0.11)
Higher sustainability–legitimacy framing bundle (higher = 1)	0.13 (0.12)	0.21 (0.12)	0.19 (0.12)	0.31 *** (0.08)	0.30 *** (0.08)	0.18 (0.11)
Direct brochure interaction: sensory richness × sustainability–legitimacy framing bundle	0.15 (0.15)	−0.26 (0.16)	0.33 * (0.16)			0.25 (0.15)
Temporal transportation				0.19 *** (0.04)	0.19 *** (0.04)	0.19 *** (0.04)
Nostalgic affect (lower-frame simple slope in interaction specifications)					0.04 (0.05)	0.05 (0.05)
Increment under higher sustainability–legitimacy framing: nostalgic affect × framing interaction					0.21 ** (0.08)	0.18 * (0.08)
R^2^	0.09	0.06	0.06	0.09	0.12	0.12
N	640	640	640	640	640	640

Note: Entries are OLS coefficients with HC3 robust SEs in parentheses. * *p* < 0.05, ** *p* < 0.01, *** *p* < 0.001. Outcomes and continuous scale predictors were z-standardized; binary condition and gender indicators remained 0/1, so binary-term coefficients are standardized-outcome contrasts rather than fully standardized slopes. Models A–C include the assigned sensory and sustainability indicators, their product, z-standardized age, and Female/Other gender indicators (male reference). Model C includes the assigned-condition interaction, so its printed sensory and sustainability coefficients are reference-category simple contrasts. The Model D specifications add within-session temporal transportation, nostalgic affect, or both. Brochure terms remain randomized contrasts, whereas coefficients linking post-treatment states to authenticity appraisal are adjusted within-session associations conditional on assignment. When the nostalgic-affect × framing interaction is included, the printed nostalgic-affect coefficient is the lower-frame simple slope. Blank cells indicate omitted terms. The aligned AU1–AU3 sensitivity appears in Supplementary Table S7.

In Model D, the brochure terms remain randomized contrasts, while the TT, NA, and authenticity coefficients are within-session associations conditional on treatment. Temporal transportation remained positive and nearly unchanged in the reduced model (β = 0.19, *p* < 0.001), the full model (β = 0.19, *p* < 0.001), and the interaction-inclusive robustness model (β = 0.19, *p* < 0.001; Table 5). The higher sustainability-framing term remained positive in the reduced and full adjusted models (β = 0.31, *p* < 0.001; β = 0.30, *p* < 0.001). When estimated alongside TT and NA, the reintroduced direct brochure interaction was imprecise (β = 0.25, *p* = 0.101). It remained imprecise in the AU1–AU3 interaction-inclusive sensitivity (β = 0.20, *p* = 0.182; Supplementary Table S7).

Nostalgic affect depended on context. In the full model, the printed NA coefficient is the lower-frame simple slope and was not independently reliable (β = 0.04, *p* = 0.485). The NA × sustainability-framing term was positive (β = 0.21, *p* = 0.009), and the corresponding higher-frame simple slope was positive (β = 0.25, *p* < 0.001; Supplementary Table S15). The same pattern held in the interaction-inclusive robustness model (NA × sustainability-framing β = 0.18, *p* = 0.023). A parallel TT × sustainability-framing term was not statistically reliable (β = −0.08, *p* = 0.320; Supplementary Table S7). The corresponding direct exploratory contrast again favored the nostalgic-affect term (Δβ = 0.29, *p* = 0.006; reported in Supplementary Table S7; archived output: outputs/study2_authenticity_specificity_check.csv), with the same non-confirmatory framing.

Conditional Wald tests showed that the TT coefficient exceeded the NA simple slope under lower-legitimacy framing in the full and interaction-inclusive models (Δβ = 0.15, *p* = 0.009; Δβ = 0.13, *p* = 0.024) and in the TT2-exclusion sensitivity (Δβ = 0.14, *p* = 0.014). Under higher-legitimacy framing, the two coefficients did not differ (Supplementary Table S15). The TT–authenticity association recurred across the central specifications, whereas the NA association was conditional on framing; this does not establish a general hierarchy between the two states. H5 therefore receives partial/conditional support and H6 qualified support for the sustainability-qualified pattern.

### 4.5. Bounded Cross-Context Synthesis

Across the two related settings, sensory cueing was positively related to temporal transportation and nostalgic affect. In Study 1, perceived sensory staging was positively associated with both states, and in Study 2 richer brochure wording increased both under random assignment. Temporal transportation was positively associated with authenticity appraisal in both studies. These recurring relations support H1, H3, and H4 without implying a general hierarchy among them.

The H5/H6 pattern is conditional. Nostalgic affect was not uniformly associated with authenticity appraisal. Instead, it became more authenticity-relevant when the sustainability context was more favorable, captured as perceived sustainability legitimacy in Study 1 and as a higher/lower sustainability-legitimacy framing bundle in Study 2. The Study 1 field evidence is a conditional association, and the Study 2 lower-frame simple slope was not independently reliable. On balance, H5 is best read as partial/conditional support, and H6 as qualified support for a stronger NA–authenticity link under more favorable sustainability conditions.

The remaining results refine the scope of the main finding. H2 received strong support in the live-setting test, where narrative coherence and participation architecture were measured directly. The brochure design did not evaluate that cue structure, so the result remains context-specific. The direct Study 2 brochure interaction is likewise a pattern confined to one experimental design. Finally, the memorability, place-attachment, advocacy, and attendance-intention models were directionally consistent but remain exploratory nomological extensions reported in Supplementary Tables S19 and S20. Table 6 summarizes the bounded cross-context synthesis across the two studies.

## 5. Discussion

### 5.1. Core Findings and Theoretical Implications

Across the two settings, sensory cueing was positively related to both temporal transportation and nostalgic affect. In Study 1, perceived sensory staging was positively associated with both states; in Study 2, the assigned high-sensory description increased both. Temporal transportation was positively associated with visitor-perceived authenticity appraisal in both studies. On RQ1, that association recurred across settings, whereas the nostalgic-affect association recurred only with a consistent legitimacy qualification (Table 6).

The findings also clarify the role of nostalgic affect. Sensory staging was positively related to nostalgic affect in the field and increased it under randomized brochure assignment. Nostalgic affect was positively associated with authenticity appraisal on average in Study 1 and under the higher sustainability–legitimacy frame in Study 2. The relationship was not uniform across contexts. This pattern fits the proposed diagnosticity account (Greifeneder et al., 2011; Pham, 1998; Schwarz & Clore, 1983): affect can inform judgment when it appears relevant to the target, while contextual cues can change the weight given to that feeling. We did not measure the attribution process itself, so this reading is qualified and awaits direct measurement in future work. It is a plausible account consistent with the observed pattern.

### 5.2. Conditional Findings and Interpretive Boundaries

The Study 1 H6 result is a conditional association measured after authenticity appraisal, so it leaves open whether legitimacy screened the use of nostalgia in time. The parallel TT × legitimacy checks were not statistically reliable in either study (Table S7). They are exploratory specificity evidence, and a temporal-transportation interaction remains possible in other settings.

Study 2 provides causal evidence only for the assigned brochure-description contrasts, which identify effects of the bundles described in Section 3.2.1 on post-vignette reports. Relationships among temporal transportation, nostalgic affect, authenticity appraisal, and attendance intention remain within-session associations conditional on assignment.

### 5.3. Exploratory Nomological Extensions and Practical Implications

We examined memorability, place attachment, advocacy, and attendance intention as exploratory nomological extensions. Their directionally consistent associations show that the focal appraisal connects with practically relevant visitor-response domains, although concurrent measurement leaves their ordering open. For practice, the Study 1 associations identify sensory specificity, coherent historical narration, and participation opportunities as candidate design features rather than demonstrated causal levers. External communication research favors transparent, evidence-based practice (Font et al., 2025; Koch & Denner, 2025; Papagiannakis et al., 2024), but Study 2 supports only the combined higher-versus-lower framing bundle and does not isolate transparency, audit, emissions-management, conservation, community-benefit, or symbolic-practice components.

### 5.4. Limitations and Future Directions

#### 5.4.1. Sampling and Generalizability

Study 1 used a nonprobability volunteer sample. Participation depended on being approached or encountering a QR prompt, choosing an uncompensated web survey, using one’s own mobile device, and completing an English-language questionnaire in multilingual event settings. These requirements may overrepresent visitors who were more engaged with the events, more comfortable with mobile surveys, or more confident in English. The study records the recruitment flow but has no data that establish how nonparticipants differed from participants. The adjusted associations remain informative about covariation among the measured perceptions and judgments. Volunteer selection, nonresponse, and unmeasured confounding limit wider inference and rule out population estimates (Huang & Shlomo, 2024; Smith et al., 2025; Velez et al., 2024).

The four field settings broaden the empirical context but do not constitute a representative sample of historic automotive events. Event fixed effects absorb mean differences among the observed settings; with four events, they cannot support rich event-level inference. The cue measures are attendee judgments and were not paired with an independent audit of event design. Study 2 has a different sampling boundary: Prolific respondents were an opt-in UK panel evaluating a generic brochure, not live attendees or a probability sample of UK adults. Both studies use one-session self-reports, and the evidence is specific to historic automotive events and descriptions of them.

#### 5.4.2. Design, Measurement Order, and Alternative Explanations

The fixed measurement order limits process claims. In Study 1, sustainability legitimacy followed authenticity appraisal and the exploratory extensions, so the H6 interaction is suggestive rather than temporally ordered. In Study 2, the randomized descriptions preceded the outcome measures, but temporal transportation, nostalgic affect, authenticity appraisal, and attendance intention were measured once in a fixed sequence. The manipulation diagnostics followed these outcomes. The randomized condition contrasts are therefore distinct from the adjusted post-treatment associations; neither study identifies a complete causal pathway or mediation process.

The two Study 2 contrasts are bundled. The sensory manipulation changes vividness and descriptive detail together with sensory content. The sustainability–legitimacy contrast combines reporting transparency, independent audit, emissions-management, heritage-conservation, and community-benefit cues. Its lower level contains skeptical cues about unclear reporting and symbolic practice. The findings cannot isolate which component produced a condition difference or be interpreted as evidence of verified environmental performance. Common evaluation tendencies, imaginability, and fluency remain plausible alternative explanations for same-session relationships (Kock et al., 2021; Milshtein et al., 2025).

Neither study was preregistered. The project-specific temporal-transportation scale was not administered alongside generic presence, immersion, or mental-time-travel measures. Visitor-perceived authenticity appraisal combines genuineness, historical fidelity, and credibility-adjacent content. In Study 2, average variance extracted for temporal transportation and nostalgic affect was marginally below 0.50, with 90% bootstrap intervals [0.447, 0.522] and [0.440, 0.519], respectively (Supplementary Table S22). The robust-fit and HTMT sensitivities strengthen fit and within-project separation evidence but do not erase mixed convergent evidence. The item sensitivities reduce dependence on particular items; the construct-scope limits remain. Future research should use probability-based or systematically stratified visitor recruitment where feasible, add nonparticipant information, and independently audit event cues. It should also separate the components of the brochure manipulations, randomize or temporally separate proposed mechanisms, and include behavioral or externally validated outcomes.

## 6. Conclusions

We asked how event cues relate to two visitor states, temporal transportation and nostalgic affect. We then asked how those states relate to visitor-perceived authenticity appraisal when the facts cannot be checked on the spot. In the field study, perceived cue domains were associated with temporal transportation. In the brochure experiment, the assigned high-sensory description raised it. Temporal transportation was positively associated with authenticity appraisal in both settings. Nostalgic affect was also linked to authenticity appraisal, more strongly so under a favorable sustainability–legitimacy context.

The claims are scoped to the designs. Study 1 provides adjusted field associations. Study 2 identifies effects of assigned brochure descriptions. Limited verifiability was a shared context, not a measured mechanism. Because Study 2 convergent evidence for temporal transportation and nostalgic affect was mixed, the cross-setting synthesis remains provisional within-project evidence. Within those limits, the results support a cautious cue-based account of authenticity appraisal.

For researchers, the findings separate past-oriented experiential presence from nostalgic affect. They also mark a context boundary on the use of nostalgia in authenticity appraisal. Wider claims await replication under the conditions in Section 5.4. For practitioners, the Study 1 associations identify historically coherent sensory, narrative, and participatory cues as candidates for further causal testing. The Study 2 result applies only to the combined higher-versus-lower sustainability–legitimacy framing bundle and does not isolate any message component.

## Figures and Tables

**Table 1:** Focal measurement evidence across studies.

**Panel A. Reliability, Convergent Validity, and Loading Ranges.**									
**Study**	**Construct**		**Items**	**M**	**SD**	**Alpha**	**CR**	**AVE/Loading Range**	
Study 1	Temporal transportation		4	5.08	1.33	0.841	0.841	0.571/0.720–0.793	
Study 1	Nostalgic affect		4	5.23	1.30	0.837	0.838	0.563/0.745–0.756	
Study 1	Visitor-perceived authenticity appraisal		4	5.15	1.31	0.823	0.824	0.539/0.682–0.771	
Study 2	Temporal transportation		4	5.45	1.13	0.790	0.790	0.485/0.681–0.706	
Study 2	Nostalgic affect		4	5.60	1.11	0.785	0.786	0.479/0.668–0.717	
Study 2	Visitor-perceived authenticity appraisal		4	5.43	1.27	0.840	0.840	0.568/0.729–0.777	
**Panel B. Overall Fit, Discriminant Validity, and Comparative Evidence.**									
**Study**	**N**	**Chi-Square (df)**	**CFI**	**TLI**	**RMSEA [90% CI]**		**SRMR**	**Max HTMT**	**Delta BIC: One Factor/Min Collapsed**
Study 1	520	49.36 (51)	1.000	1.000	0.000 [0.000, 0.027]		0.020	0.428	1088.44/510.32
Study 2	640	53.91 (51)	0.999	0.998	0.009 [0.000, 0.028]		0.028	0.284	1232.60/602.18

Note. Alpha = Cronbach’s alpha; CR = composite reliability; AVE = average variance extracted; HTMT = heterotrait–monotrait ratio; RMSEA confidence intervals are 90%. Delta BIC values compare the stated alternative with the three-factor model; positive values favor the three-factor model. Pairwise-collapsed refers to global 12-item two-factor alternatives that collapsed TT with NA, TT with authenticity appraisal, or NA with authenticity appraisal. Full item loadings and focused pairwise comparisons appear in the Supplementary Materials.

**Table 2:** Correlations among core and extension constructs (Study 1).

Construct	SS	TT	NA	AU	MEM	PA	ADV	SL
Sensory staging	1.00	0.50	0.51	0.24	0.26	0.19	0.18	0.12
Temporal transportation	0.50	1.00	0.32	0.36	0.33	0.24	0.25	0.12
Nostalgic affect	0.51	0.32	1.00	0.23	0.34	0.23	0.14	0.13
Authenticity appraisal	0.24	0.36	0.23	1.00	0.15	0.39	0.34	0.18
Memorability	0.26	0.33	0.34	0.15	1.00	0.23	0.19	0.01
Place attachment	0.19	0.24	0.23	0.39	0.23	1.00	0.35	0.03
Advocacy intentions	0.18	0.25	0.14	0.34	0.19	0.35	1.00	0.04
Perceived sustainability legitimacy	0.12	0.12	0.13	0.18	0.01	0.03	0.04	1.00

Note: Pearson correlations (two-tailed). SS = sensory staging; TT = temporal transportation; NA = nostalgic affect; AU = authenticity appraisal; MEM = memorability; PA = place attachment; ADV = advocacy intentions; SL = perceived sustainability legitimacy. Extension variables are included for completeness.

**Table 3:** Study 1 adjusted field associations among cue perceptions, experiential states, and authenticity appraisal.

	Model 1: TT	Model 2: NA	Model 3: Authenticity Appraisal
Sensory staging	0.35 *** (0.04)	0.40 *** (0.04)	
Narrative coherence	0.22 *** (0.04)	0.17 *** (0.04)	
Participation architecture	0.14 *** (0.04)	0.05 (0.04)	
Personal nostalgia proneness	0.03 (0.04)	0.13 *** (0.04)	
Historical nostalgia proneness	0.13 *** (0.04)	0.15 *** (0.04)	
Temporal transportation			0.30 *** (0.04)
Nostalgic affect			0.14 *** (0.04)
Perceived sustainability legitimacy			0.13 ** (0.04)
Nostalgic affect × perceived sustainability legitimacy			0.18 *** (0.04)
R^2^	0.34	0.34	0.20
N	520	520	520

Note: Entries are standardized coefficients β with HC3 robust SEs in parentheses. * *p* < 0.05, ** *p* < 0.01, *** *p* < 0.001. Study 1 cue terms are respondent perceptions, and the sustainability–legitimacy term is a later respondent judgment from the same fixed-order field survey, so the coefficients are adjusted field associations. The interaction term is a conditional association measured later in the questionnaire. Supplementary Tables S7 and S15 report the corresponding conservative SS1–SS3 no-overlap and TT2-exclusion sensitivities, which preserve the focal cueing → TT and TT → authenticity patterns.

**Table 4:** Study 2 unadjusted cell means (SD) for focal outcomes and diagnostic composites across the four brochure conditions.

Condition	Temporal Transportation	Nostalgic Affect	Authenticity Appraisal	Diagnostic: Perceived Sensory Richness	Diagnostic: Perceived Sustainability Legitimacy
Low sensory × lower-legitimacy framing bundle	5.07 (1.21)	5.22 (1.17)	5.12 (1.30)	4.98 (0.83)	4.44 (0.77)
Low sensory × higher-legitimacy framing bundle	5.22 (1.20)	5.45 (1.12)	5.35 (1.30)	5.01 (0.73)	5.69 (0.69)
High sensory × lower-legitimacy framing bundle	5.60 (0.98)	5.88 (0.97)	5.30 (1.26)	5.91 (0.57)	4.49 (0.87)
High sensory × higher-legitimacy framing bundle	5.92 (0.91)	5.83 (1.02)	5.96 (1.06)	5.99 (0.57)	5.76 (0.60)

Note: Entries are unadjusted cell summaries (n = 160 per cell); conditions are the bundled brochure descriptions defined in Section 3.2.1. The randomized cue-to-state contrasts were 0.61 [0.44, 0.78] for temporal transportation and 0.52 [0.35, 0.69] for nostalgic affect. The largest observed overall authenticity contrast was 0.45 [0.25, 0.64], and the largest observed simple contrast was concentrated in the high-sensory cell (0.66 [0.40, 0.92]).

**Table 6:** Bounded cross-context synthesis of the cue-to-state-to-authenticity pattern across the two study settings.

Claim Cluster/Display	Synthesis Layer	Relation Summarized	Study 1 (Post-Event Field)	Study 2 (Brochure-Based)	Bounded Cross-Context Reading
Cross-context recurrence across the two related settings					
Sensory cueing → temporal transportation (H1)	Cross-context recurrence	Stronger sensory cueing aligned with stronger temporal transportation	Positive adjusted field association	Positive randomized brochure-condition contrast	The positive H1 relation recurred across both settings.
Temporal transportation → authenticity appraisal (H4)	Cross-context recurrence	Temporal transportation was positively associated with authenticity appraisal in both study settings	Positive adjusted field association	Consistent positive adjusted within-session association conditional on treatment across Model D specifications	The positive H4 association recurred across both settings; this is not temporal stability.
Sensory cueing → nostalgic affect (H3)	Cross-context recurrence	Stronger sensory cueing also aligned with stronger nostalgic affect	Positive adjusted field association	Positive randomized brochure-condition contrast	The positive H3 relation recurred across both settings.
Conditional/context-bounded patterns					
Context-qualified nostalgic-affect route (H5/H6)	Conditional/context-bounded support	Nostalgic affect became more authenticity-relevant under more favorable sustainability conditions	Positive at average/higher legitimacy; later-measured conditional association	Higher-frame simple slope positive; lower-frame simple slope not independently reliable	H5 received partial/conditional support, and H6 received qualified support.
Localized direct brochure authenticity difference	Study 2 boundary, not a cross-study headline	Direct authenticity shift was concentrated in the favorable brochure combination	Not applicable in the field design	Localized to the high-sensory/higher-legitimacy cell; weaker in fuller and AU1–AU3 sensitivity specifications	This localized Study 2 pattern should not carry the main cross-study inference.
Context-specific or exploratory extensions					
Live-setting cue structure (H2)	Context-specific support	Narrative coherence and participation architecture refined TT beyond sensory staging	Positive adjusted field association	Not tested in the brochure design	H2 received strong support in the live-setting test, but not cross-study replication.
Exploratory nomological extensions	Exploratory extension	Memorability, place attachment, advocacy, and attendance intention	Directionally consistent exploratory associations reported in Table S19	Directionally consistent exploratory associations reported in Table S20	These same-session results extend nomological scope but do not establish temporal or causal ordering.

Note: Rows separate cross-context recurrence, conditional/context-bounded patterns, and context-specific or exploratory extensions. Study 1 entries summarize adjusted field associations. In Study 2, direct brochure terms identify randomized brochure-condition contrasts, whereas rows involving TT, NA, or authenticity appraisal summarize adjusted within-session associations conditional on treatment. Sustainability legitimacy is measured later in Study 1 and represented by a higher/lower framing bundle in Study 2; accordingly, H5 is summarized as partial/conditional support and H6 as qualified support.

## Data Availability

De-identified data and reproducibility materials are publicly available on Zenodo at https://doi.org/10.5281/zenodo.22214518 (DOI: 10.5281/zenodo.22214518). The deposit includes the analysis-ready CSV datasets used in the article, executable code, machine-readable outputs, retained seed assets required to rebuild tables and figures, and the de-identified source materials and documentation needed to audit or rerun the analyses. The README and MANIFEST files within the deposit document package structure, run order, and table/figure mappings.

## Supplementary Materials

The following supporting information can be downloaded at: https://www.mdpi.com/article/10.3390/bs16091644/s1. The supporting information comprises behavsci-16-01644-supplementary.pdf (Sections S0–S5) and the consolidated workbook behavsci-16-01644-supplementary-tables.xlsx. The PDF contains Table S2.1: Global fit of the focal three-factor CFA and the one-factor alternative; Table S2.2: Comparative models: collapsed and one-factor alternatives against the preferred specification; Table S2.3: Standardized item loadings and residual variances from the focal three-factor CFAs; Table S2.4: Reliability, convergent validity, and loading ranges for the focal constructs; Table S2.5: Latent factor correlations, HTMT, and observed scale correlations for the focal construct pairs; Table S2.6: Multi-start convergence summary for the focal CFA models; Table S3.1: Reduced latent-regression reparameterization (values reproduced from Table S16). These PDF tables are also reproduced on the workbook’s PDF_Tables worksheet. The workbook contains the following tables, with worksheets indexed in its Contents sheet and in the PDF: Table S1. AVE and composite reliability (Study 1 focal constructs and cue block). Table S2. Study 1 factor loadings and factor-model diagnostics. Table S3. Study 1 seven-factor and one-factor model fit. Table S4. Study 1 item-level descriptive statistics and diagnostics. Table S5. Study 2 item-level descriptive statistics and diagnostics. Table S6. Descriptive summaries for focal Study 2 measures and diagnostic composite summaries. Table S7. Robustness checks for main coefficients (Studies 1 and 2). Table S8. TT, nostalgic affect, and authenticity appraisal: within-project measurement support and factor-model comparisons. Table S9. Study 2 manipulation-targeting and item-level diagnostic contrasts. Table S10. Study 2 randomized brochure contrasts for focal outcomes and diagnostic composite summaries (unadjusted mean differences with 95% CIs). Table S11. Study 2 recruitment and exclusion flow. Table S12. Study 2 condition-level randomization, attrition, and quality-control counts. Table S13. Study 2 completed-sample robustness comparison. Table S14. Study 2 demographic descriptors by experimental cell (final analytic sample). Table S15. Exact coefficient reporting for focal models and summary indices. Table S16. Reduced latent-variable reparameterization of the TT–NA–authenticity-appraisal core block. Table S17. Bounded cross-context synthesis of the cue-to-state-to-authenticity pattern across the two study settings. Table S18. Integrated Study 2 cell means, key randomized brochure-condition contrasts, and the direct randomized authenticity-appraisal condition model. Table S19. Secondary exploratory nomological analyses in Study 1: models and indirect-association summaries. Table S20. Secondary exploratory nomological analyses in Study 2: attendance-intention model and association-chain summaries. Table S21. Study 1 secondary-scale reliability and descriptive statistics. Table S22. Robust measurement sensitivity for the focal three-factor models.

## Abbreviations

The following abbreviations are used in this manuscript:

α	Cronbach’s Alpha
AVE	Average Variance Extracted
AU	Visitor-perceived authenticity appraisal
CFA	Confirmatory Factor Analysis
CI	Confidence Interval
CR	Composite Reliability
EFA	Exploratory Factor Analysis
HC3	Heteroskedasticity-Consistent Standard Errors, Type 3
HTMT	Heterotrait–Monotrait Ratio
IHU	International Hellenic University
NA	Nostalgic Affect
OLS	Ordinary Least Squares
SD	Standard Deviation
SEs	Standard Errors
SS	Sensory Staging
TT	Temporal Transportation
UK	United Kingdom
