# Supplementary material for: Cue-Based Authenticity Appraisals in Limited-Verifiability Contexts: A Two-Study Test in Historic Automotive Events
Source: Behav Sci (Basel). 2026 Sep 14;16(9):1644. doi: 10.3390/bs16091644 (PMC13603197; doi:10.3390/bs16091644)
Supplement: Supplementary file 1 [file behavsci-16-01644-s001.zip › behavsci-4311859-supplementary.pdf]

Supplementary Materials

For: "Cue-Based Authenticity Appraisals in Limited-Verifiability Contexts: A Two-Study Test in Historic Automotive Events"

S0. Overview

This appendix documents the technical materials that support the manuscript's bounded cue-to-state-to-authenticity-appraisal argument across two related settings: post-experience field evaluation in Study 1 and brochure-based evaluation of described materials in Study 2. Limited verifiability is the shared judgment context, not an experimental factor. Temporal transportation and nostalgic affect are the experiential states; visitor-perceived authenticity appraisal is the focal evaluative judgment; sustainability legitimacy functions as a contextual qualifier of the nostalgic-affect route; and memorability, place attachment, advocacy, and attendance intention are retained only as exploratory nomological extensions. The appendix does not extend the manuscript's main claims. Instead, it records procedures, item wording, measurement diagnostics, and robustness checks that document how the reported findings were evaluated and where their interpretation remains bounded. Sections S0.1–S0.3 cover design details, sample flow, and construct boundaries; S1 reproduces the measures; S2 reports the focal measurement validation and psychometric diagnostics for the within-project uses of the measures; S3 reports robustness and sensitivity analyses; and S4 reproduces the Study 2 stimuli.

Guide to supplementary contents: The table below maps major technical topics to the supplement sections, tables, and related display files most relevant to each topic.

Guide to supplementary contents

**Topic**	**Supplement location(s)**
Measures and item wording	S1.1–S1.2; see S4.1–S4.3 for the Study 2 brochure text.
Focal measurement validation (CFA fit, loadings, reliability, HTMT, comparative models)	S2.1–S2.4 and Tables S2.1–S2.5; Tables S1, S6, and S8; manuscript Table 1.
Psychometrics and construct separation	S2.1-S2.7; Tables S1, S2, S6, S8, S16, S21-S22.
Sensitivity checks for core claims	S3.1-S3.4; Tables S7, S13, S15, and S22.
Exact coefficients, CIs, and conditional TT-versus-NA comparisons	Table S15; see Table S7 for linked TT2-exclusion, AU1–AU3, conservative no-overlap, and specificity rows.
Cross-context synthesis and integrative summary displays	S3.5; Table S17; Tables S18–S20; manuscript Tables 4–6.
Study 2 brochure wording and manipulated text	S4.1–S4.3.
Manipulation checks and condition integrity	S1.2; Tables S9–S10; Tables S12–S14.
Sample flow, exclusions, and completed-sample sensitivity	S0.1–S0.2; Tables S11–S14.
Conservative SS1–SS3 no-overlap check for H1	S1.1 (SS and TT item wording); S3.1; Table S7.
Event heterogeneity / leave-one-event-out robustness	S0.1; S3.1; Table S7.

Table number and caption	Worksheet tab(s)
Table S2. Study 1 factor loadings and factor-model diagnostics.	S2_EFA_Loadings<br>S2_Oblimin_Summary<br>S2_Oblimin_Loadings<br>S2_Oblimin_FactorCorr<br>S2_FocalCFA_Loadings<br>S2_FocalCFA_FactorCorr<br>S2_CueBlock_Fit<br>S2_CueBlock_Loadings<br>S2_CueBlock_FactorCorr
Table S3. Study 1 seven-factor and one-factor model fit.	S3_ModelFit
Table S4. Study 1 item-level descriptive statistics and diagnostics.	S4_ItemStats_Study1
Table S5. Study 2 item-level descriptive statistics and diagnostics.	S5_ItemStats_Study2
Table S6. Descriptive summaries for focal Study 2 measures and diagnostic composite summaries.	S6_Reliability
Table S7. Robustness checks for main coefficients (Studies 1 and 2).	S7_Robustness<br>S7_EventAnalyses<br>S7_Diagnostics
Table S8. TT, nostalgic affect, and authenticity appraisal: within-project measurement support and factor-model comparisons.	S8_Summary<br>S8_Global_CFA_Fit<br>S8_Pairwise_Comparisons<br>S8_Loadings_Study1<br>S8_FactorCorr_Study1<br>S8_Loadings_Study2<br>S8_FactorCorr_Study2<br>S8_HTMT
Table S9. Study 2 manipulation-targeting and item-level diagnostic contrasts.	S9_ManipulationChecks
Table S10. Study 2 randomized brochure contrasts for focal outcomes and diagnostic composite summaries (unadjusted mean differences with 95% CIs).	S10_MainEffects<br>S10_SimpleEffects
Table S11. Study 2 recruitment and exclusion flow.	S11_Recruitment
Table S12. Study 2 condition-level randomization, attrition, and quality-control counts.	S12_ConditionIntegrity
Table S13. Study 2 completed-sample robustness comparison.	S13_CompletedSample
Table S14. Study 2 demographic descriptors by experimental cell (final analytic sample).	S14_Demographics
Table S15. Exact coefficient reporting for focal models and summary indices.	S15_Study1_Models<br>S15_Study2_Models<br>S15_Study1_Indirect<br>S15_Study2_AssocChain<br>S15_TT_vs_NA<br>S15_AU123_Sensitivity
Table S16. Reduced latent-variable reparameterization of the TT–NA–authenticity-appraisal core block.	S16_LatentModels
Table S17. Bounded cross-context synthesis of the cue-to-state-to-authenticity pattern across the two study settings.	S17_CrossStudy

Table number and caption	Worksheet tab(s)
Table S18. Integrated Study 2 cell means, key randomized brochure-condition contrasts, and the direct randomized authenticity-appraisal condition model.	S18_IntegratedResults
Table S19. Secondary exploratory nomological analyses in Study 1: models and indirect-association summaries.	S19_Study1_Models<br>S19_Study1_Indirect
Table S20. Secondary exploratory nomological analyses in Study 2: attendance-intention model and association-chain summaries.	S20_IntentionModel<br>S20_AssocChain
Table S21. Study 1 secondary-scale reliability and descriptive statistics.	S21_SecondaryReliability
Table S22. Robust measurement sensitivity for the focal three-factor models.	S22_RobustMeasurement

Institutional Review Board Statement: Both studies were conducted in accordance with the Declaration of Helsinki and were approved by the Ethics Committee of the Organizations Management, Marketing and Tourism, IHU (protocol code 162/08-1-2024; approval date 08-01-2024).

S0.1 Study 1 procedural details and analytic sample flow

Study 1 fieldwork took place across the 2024 editions of four European historic automotive events: Rallye Monte-Carlo Historique (1–4 February 2024; Monaco start and French route towns), Historic Acropolis Rally (6–9 June 2024; Athens ceremonial start and stages in Central Greece), Mille Miglia (11–14 June 2024; Brescia–Rome–Brescia route, excluding the final ceremonial finish day), and Goodwood Revival (6–8 September 2024; Goodwood Motor Circuit, West Sussex). Recruitment followed a standardized site-intercept plus QR protocol. Research assistants worked in high-footfall but low-congestion areas where attendees naturally paused, including start villages, service-park pedestrian corridors, paddock perimeter entrances, food-court seating edges, route control points, and public viewing corridors. Recruiters used a brief neutral invitation and targeted the two focal attendee roles – drivers/co-drivers and spectators. The survey was administered in English only; there was no language selector, although recruiters could use a brief local-language invitation before directing respondents to the English questionnaire.

Because participation was voluntary and uncompensated in multilingual field settings, the resulting sample is a self-selected attendee subset, likely favoring respondents comfortable with an English-language questionnaire and somewhat more internationally oriented, time-available, or highly engaged. Respondents completed the mobile-optimized survey on their own devices after consent. Recruiter assistance was limited to QR access; staff did not read questions aloud, interpret item wording, or influence responses. The programmed flow was consent; age/role eligibility items; event-context questions; perceived cue judgments; temporal transportation plus attention check AC1; nostalgic affect; authenticity appraisal and memorability; place attachment and advocacy; sustainability legitimacy; nostalgia-proneness items plus attention check AC2; and optional demographics. Items were randomized within subscales, but block order was fixed. Because the sustainability-legitimacy block followed authenticity appraisal, memorability, place attachment, and advocacy, the Study 1 interaction is later-measured within-session evidence.

Across all four events, 1,710 attendees were approached, 715 opened the survey link or scanned the QR code, 620 consented and started the questionnaire, 57 submitted partial responses, and 563 submitted complete responses. The final analytic sample (N = 520) followed the archived cleaning rules: under 18 (n = 2), ineligible role (n = 7), duplicate submission (n = 6), failed attention check (n = 10), straightlining or other low-quality responding (n = 12), and missing key variables (n = 6). Event-specific flow was as follows: Mille Miglia—420 approached, 163 started, 148 completed, 136 analytic; Goodwood Revival—470 approached, 165 started, 153 completed, 143 analytic; Rallye Monte-Carlo Historique—380 approached, 134 started, 120 completed, 111 analytic; Historic Acropolis Rally—440 approached, 158 started, 142 completed, 130 analytic. Duplicate submissions were defined as repeated DeviceID + IPAddress combinations within the same event window, with the earliest complete record retained. Straightlining or other low-quality responding was defined as near-zero within-respondent variance across the core Likert blocks (SD < 0.15 across SS–ADV items) or a complete submission recorded in under 60 seconds; borderline automated flags

were manually checked before exclusion. Missing key variables were defined as missing all items for at least one key main-state block (TT or NA) or missing more than 50% of the core construct items.

The Study 1 cue variables analyzed in the manuscript therefore represent respondent perceptions of event features, not independent field audits. Sustainability legitimacy was likewise a later respondent judgment collected in the same survey session, so the Study 1 interaction and association-chain summaries are within-session field-association evidence. Given only four events, the manuscript's main models use event fixed effects as a pragmatic adjustment for between-event mean differences, which is all that four events can support. The companion by-event sheet in Table S7 reports event-by-role counts and core demographics for this final analytic sample.

S0.2 Study 2 procedural details and analytic sample flow

Study 2 was a brochure-style vignette experiment fielded online in Qualtrics between 14 and 17 October 2025 and recruited through Prolific's opt-in UK adult panel. Recruitment targeted one response per participant from adults aged 18–70 currently resident in the United Kingdom, and the survey was administered in English. The survey was advertised as taking 8 minutes and paid GBP 1.50 through participants' Prolific balances, with no bonus payment. The programmed flow was consent; age and country eligibility screens; Qualtrics Randomizer (1:1:1:1 allocation to brochure descriptions crossing described sensory richness and a higher/lower sustainability-legitimacy framing bundle); a condition-specific brochure description; one fixed post-vignette measurement sequence (TT, NA, AU, INT); manipulation-diagnostic items; attention check; demographics; and debrief. Brochure wording was randomized. The sensory wording bundled vividness and multisensory imagery. The higher sustainability-legitimacy framing bundled reporting transparency, independent audit, emissions-management, heritage-conservation, and community-benefit cues, whereas the lower framing contained skeptical cues about unclear reporting and symbolic practice; it was not an extreme denunciation. Each level of the framing bundle is therefore a bundled description. The design therefore identifies brochure-description effects on post-vignette self-reports about described materials. Because TT, NA, AU, and INT were all measured once in that same post-vignette sequence, coefficients linking them are within-session associations conditional on the randomized brochure terms.

Across the field period, 812 panel members were invited, 708 opened the survey link, and 688 consented and met eligibility criteria; all 688 eligible respondents were randomized (172 records per cell before quality control). Twenty-eight randomized respondents submitted partial surveys and 660 completed the questionnaire. Median completion time among finished respondents was 462 seconds (7.7 minutes). After applying the documented post-completion quality-control rules to the 660 finished records—duplicate completed records (n = 5), attention-check failure (n = 6), speeding (n = 4), straightlining or other low-quality responding (n = 3), and bot/VPN/proxy flags (n = 2) – the final analytic sample comprised 640 participants (160 per cell). The detailed recruitment/exclusion flow is reported in Table S11. Supplementary Table S14 reports descriptive age, gender, and UK-region distributions by experimental cell for the final analytic sample.

Table S11 presents Study 2 recruitment and exclusion flow. The invitation count comes from the archived recruitment record because panel invitations are not represented in the respondent-level export. All later counts were verified from the shared de-identified source export. Randomization was balanced at 172 records per cell before quality control and the final analytic sample retained 160 records per cell. No completed cases were removed for age or country ineligibility.

Table S12 presents Study 2 condition-level randomization, attrition, and quality-control counts. Counts were computed from the shared de-identified source export using RandomizerBranch and record_disposition. Completed-before-QC counts are not shown because they equal randomized minus partial/incomplete records in each cell. Archived survey-flow metadata indicate that back-end monitoring continued until each cell reached 160 valid completes, and the balanced final counts are consistent with that protocol. This table documents assignment integrity and symmetric attrition across the four cells; Tables S9-S10 document whether the assigned brochure wording selectively moved the intended diagnostic composites.

S0.3 Conceptual clarification of the focal judgment context and constructs

S0.3.1 Limited verifiability, authenticity appraisal, and adjacent concepts

Limited verifiability is the shared judgment context in this project; it is neither measured nor manipulated in either study. It refers to settings in which evaluators encounter curated, immediately observable cues but do not have decisive historical confirmation available at the moment of judgment. The issue is therefore cue-based inference under partial proof, not the absence of cues. In the present project, that context is what makes a cue-to-state-to-authenticity-appraisal account theoretically relevant: participants use staging, narrative, participation, or brochure cues to judge whether the represented past feels genuine without being able to verify that conclusion directly.

This differs from direct verifiability, where authenticity can be checked at once, and from generic uncertainty, which may arise for many reasons without defining the same authenticity problem. It also differs from visitor-perceived

authenticity appraisal itself. Limited verifiability describes the conditions under which the judgment is formed; authenticity appraisal records the evaluator's conclusion that the represented past feels genuine, credible, or experientially true. In line with the manuscript, it is a visitor-perceived appraisal, distinct from expert authentication and from existential authenticity, and broader than cue credibility or message believability. Limited verifiability is neither measured nor manipulated in either study: Study 1 observes field judgments in that shared context, whereas Study 2 randomizes brochure cueing and a higher/lower sustainability-legitimacy framing bundle within it.

S0.3.2 Temporal transportation, nostalgic affect, and sustainability legitimacy within that context

Within that judgment context, temporal transportation is used here as a project-specific label for past-oriented experiential presence: the felt temporal nearness of a past era while the evaluator remains in the present event or representation. It is therefore more specific than generic immersion or absorption and more state-like than a broad historical orientation. Nostalgic affect indexes the emotional tone of engaging with that past – warmth, wistfulness, or bittersweetness – whereas authenticity appraisal records the evaluative conclusion that the represented past feels genuine or experientially true. Narrative transportation, generic presence/immersion, and autobiographical mental time travel remain adjacent but non-identical constructs. The four-item TT block is therefore a bounded, project-specific operationalization for the present analyses.

Sustainability legitimacy is treated separately from those experiential states. It captures a contextual evaluation of whether the event's present-day sustainability stance seems appropriate, serious, and defensible enough for nostalgic affect to count as authenticity-relevant information. In Study 1 it is a later-measured respondent judgment; in Study 2 it is represented by a broader higher/lower sustainability-legitimacy framing bundle. Sections S0.3.1–S0.3.2 record the distinctions used throughout the manuscript and supplement, and Tables S7 and S16 report the robustness routes most relevant to these construct boundaries.

S1. Measures and item wording

S1.1 Study 1 (field survey)

Sensory staging (SS; 4 perceived cue-judgment items):

- SS1. The event deliberately uses sound, smell, and visuals to evoke the era.
- SS2. The sensory atmosphere (e.g., engine sounds, scents, textures) feels carefully crafted.
- SS3. The event environment feels immersive because multiple senses are engaged.
- SS4. Sensory details make the experience feel like stepping into the past.

Narrative coherence (NC; 3 perceived cue-judgment items):

- NC1. The event tells a clear story about the past it represents.
- NC2. The different parts of the event fit together into a coherent theme.
- NC3. The route/rituals make sense as part of one heritage narrative.

Participation architecture (PA; 3 perceived cue-judgment items):

- PA1. The event gives me chances to actively take part rather than only watch.
- PA2. I could interact with people/places along the route in meaningful ways.
- PA3. My involvement (watching, cheering, navigating, helping) felt like part of the event.

Temporal transportation (TT; 4-item project-specific operationalization used in this paper):

- TT1. At times during the event, I felt as if I were living in another era.
- TT2. I became absorbed in the event experience and lost track of the present.
- TT3. The event made the past feel vividly present to me.
- TT4. It felt like I was "there back then," not just observing from today.

TT2 is the item closest to generic absorption or immersion wording. The focused robustness check in S3 therefore recomputes TT from TT1, TT3, and TT4 only for the core Study 1 and Study 2 models. It is retained in the main operationalization because it still loads with the other TT items and fits the paper's bounded past-oriented experiential-presence account, but the alternative composite shows that the substantive pattern does not depend on this one item. In Study 1, the block is answered after live multisensory attendance, so it captures live post-experience temporal nearness, whereas Study 2 elicits brochure-based imagined presence.

Nostalgic affect (NA; 4 items):

- NA1. The event made me feel nostalgic.
- NA2. The event made me feel a warm longing for the past.
- NA3. The event made me feel sentimental about earlier times.
- NA4. The event made me miss the past (in a pleasant way).

Visitor-perceived authenticity appraisal (AU; 4 items):

- AU1. The experience felt authentic rather than staged.
- AU2. What I saw and felt seemed genuine.
- AU3. The event felt true to its historical character.
- AU4. The heritage presented here felt credible.

Memorability (MEM; 3 items):

- MEM1. I will remember this experience for a long time.
- MEM2. The experience feels vivid in my mind.
- MEM3. This event stands out as a memorable experience.

Place attachment (PLA; 4 items):

- PLA1. I feel an emotional bond with the place(s) associated with this event.
- PLA2. This place feels like part of who I am (even if only as a visitor).
- PLA3. I identify with the character of this place.
- PLA4. I feel attached to this destination because of the event.

Advocacy intentions (ADV; 3 items):

- ADV1. I would recommend this event/destination to others.
- ADV2. I would say positive things about this event/destination.
- ADV3. I intend to return in the future if I can.

Perceived sustainability legitimacy (SL; 3 items; Study 1 perceived contextual judgment, administered after authenticity appraisal, memorability, place attachment, and advocacy in the fixed survey order):

- SL1. The event's sustainability initiatives feel genuine, not symbolic.
- SL2. The event seems to take environmental responsibility seriously.
- SL3. The event's practices fit today's sustainability expectations.

Personal nostalgia proneness (PNP; 4 items):

- PNP1. I often feel nostalgic about my own past.
- PNP2. Thinking about my past gives me warm feelings.
- PNP3. I frequently miss aspects of my earlier life.
- PNP4. I enjoy reminiscing about my past.

Historical nostalgia proneness (HNP; 4 items):

- HNP1. I often feel nostalgic for times before I was born.
- HNP2. I sometimes long for earlier eras of society and culture.
- HNP3. I enjoy imagining what life was like in earlier times.
- HNP4. I feel drawn to the atmosphere of past decades.

S1.2 Study 2 (brochure-style vignette experiment)

Unless otherwise noted, attitudinal items in Study 2 used a 7-point agreement scale (1 = Strongly disagree, 7 = Strongly agree). Study 2 used the same item wording for TT, NA, and AU as in Study 1, preceded by the instruction that responses should be based only on the brochure description. This preserves conceptual continuity across two elicitation modes: Study 2 respondents report brochure-based imagined presence and nostalgic emotion about described materials, whereas Study 1 respondents report live post-experience states. That difference is relevant when interpreting the much weaker TT–NA coupling in Study 2. Attendance intention (INT; 3 items) was the only new substantive block. It is treated as an exploratory nomological extension. Manipulation-diagnostic items captured uptake of the randomized sensory-richness and higher/lower sustainability-legitimacy framing bundles; these diagnostics assess whether respondents read the intended brochure cues, not separate latent constructs.

- INT1. If this event were held near me, I would want to attend.
- INT2. I would recommend this event to friends who like heritage experiences.
- INT3. I would look for more information and consider registering/visiting.

Manipulation-diagnostic items capturing uptake of the randomized brochure bundles were:

- MC_SR1. The event description made the experience feel sensory-rich.
- MC_SR2. I could easily imagine the sounds/smells/feel of the event.
- MC_SR3. The description engaged multiple senses.
- MC_SL1. The event seems to manage its environmental impact credibly.
- MC_SL2. The sustainability claims seem trustworthy.

- MC_SL3. The event's sustainability approach fits today's expectations.

Post-vignette instruction: "Please indicate how much you agree or disagree with each statement, based only on the event description you just read." The instructed-response attention check was: "To show that you are paying attention, please select 'Somewhat agree' for this item." The correct response was 5 = Somewhat agree. Demographics included age (numeric entry), gender (female, male, non-binary/other, prefer not to say), and United Kingdom region (England, Scotland, Wales, Northern Ireland).

S2. Focal measurement validation and psychometric diagnostics

Before estimating the substantive regressions, we evaluated the focal measurement structure for temporal transportation, nostalgic affect, and visitor-perceived authenticity appraisal. In each study, the 12 items were fitted to a correlated three-factor confirmatory factor analysis (CFA) using covariance-based maximum likelihood, estimated directly from the shared covariance matrices. We compared that specification with a single-factor model, three global two-factor models that collapsed each pair of constructs in turn, and focused pairwise one- versus two-factor models. Tables S2.1–S2.5 report chi-square, CFI, TLI, RMSEA with its 90% confidence interval, SRMR, BIC, standardized loadings, Cronbach's alpha, composite reliability, average variance extracted (AVE), factor correlations, and HTMT, so that the measurement model can be assessed here without consulting the workbook; Three layers are kept distinct: the CFAs evaluate measurement structure; the hypothesis tests use unit-weighted scale means in HC3 OLS regressions; and Table S16 is a reduced pooled/unadjusted latent reparameterization rather than a full robustness or invariance test.

The broader Study 1 item set was evaluated with a theory-specified seven-factor maximum-likelihood loading screen of 26 items. The solution fitted well, chi-square(164) = 180.90, CFI = .997, TLI = .994, RMSEA = .014, and SRMR = .014. Oblimin rotation left all 26 primary allocations unchanged; the largest absolute secondary loading was .112 (Tables S2-S3). This was a theory-specified loading screen, not a data-driven factor-retention exercise.

The single-factor alternative in Table S3 remains a descriptive common-method screen only. Focused cue-domain CFAs are reported separately. Together with the focal CFAs, these displays report the loading, correlation, fit, and information-criterion evidence for the constructs closest to the main claims without presenting the loading screen as a substitute for formal factor-retention analysis.

S2.1 Focal three-factor CFA: fit and comparative models

The correlated three-factor model fitted the focal items well in Study 1, chi-square(51) = 49.36, CFI = 1.000, TLI = 1.000, RMSEA = .000, 90% CI [.000, .027], SRMR = .020, and in Study 2, chi-square(51) = 53.91, CFI = .999, TLI = .998, RMSEA = .009, 90% CI [.000, .028], SRMR = .028 (Table S2.1). The one-factor alternative was substantially worse (delta BIC = 1088.44 and 1232.60, respectively), as were all global pairwise-collapsed alternatives (minimum delta BIC = 510.32 and 602.18); the focused pairwise eight-item comparisons preferred the two-factor solution for every pair in both studies (Table S2.2). Positive delta BIC values favor the three-factor model. Pairwise-collapsed refers to global 12-item two-factor alternatives that collapsed TT with NA, TT with authenticity appraisal, or NA with authenticity appraisal.

Table S2.1. Global fit of the focal three-factor CFA and the one-factor alternative.

Study	N	Model	chi-square	df	CFI	TLI	RMSEA	SRMR	BIC
Study 1	520	Three-factor	49.36	51	1.000	1.000	.000	.020	218.21
Study 1	520	One-factor	1156.56	54	.542	.440	.198	.150	1306.65
Study 2	640	Three-factor	53.91	51	.999	.998	.009	.028	228.36
Study 2	640	One-factor	1305.89	54	.477	.361	.190	.173	1460.97

Note. Covariance-based maximum likelihood; RMSEA 90% confidence intervals are [.000, .027] for Study 1 and [.000, .028] for Study 2 (three-factor models). Source: P5_MODEL_FIT_SUMMARY.csv; identical values appear in Table S8.

Table S2.2. Comparative models: collapsed and one-factor alternatives against the preferred specification.

Study	Comparison class	Alternative	Preferred	Alternative chi-square (df)	Alternative BIC	Delta BIC
Study 1	global collapsed alternative	TT+NA collapsed	Three-factor	656.41 (53)	812.75	594.55
Study 1	global collapsed alternative	TT+AU collapsed	Three-factor	572.18 (53)	728.53	510.32
Study 1	global collapsed alternative	NA+AU collapsed	Three-factor	708.00 (53)	864.35	646.14
Study 1	global one-factor alternative	One factor	Three-factor	1156.56 (54)	1306.65	1088.44
Study 1	pairwise 8-item comparison	TT-NA one-factor	Two-factor	628.76 (20)	728.82	600.84
Study 1	pairwise 8-item comparison	TT-AU one-factor	Two-factor	531.58 (20)	631.64	516.17
Study 1	pairwise 8-item comparison	NA-AU one-factor	Two-factor	653.85 (20)	753.91	622.96

Study	Comparison class	Alternative	Preferred	Alternative chi-square (df)	Alternative BIC	Delta BIC
Study 2	global collapsed alternative	TT+NA collapsed	Three-factor	748.61 (53)	910.15	681.78
Study 2	global collapsed alternative	TT+AU collapsed	Three-factor	669.01 (53)	830.55	602.18
Study 2	global collapsed alternative	NA+AU collapsed	Three-factor	689.13 (53)	850.66	622.30
Study 2	global one-factor alternative	One factor	Three-factor	1305.89 (54)	1460.97	1232.60
Study 2	pairwise 8-item comparison	TT-NA one-factor	Two-factor	695.22 (20)	798.60	673.08
Study 2	pairwise 8-item comparison	TT-AU one-factor	Two-factor	623.47 (20)	726.85	607.59
Study 2	pairwise 8-item comparison	NA-AU one-factor	Two-factor	668.44 (20)	771.83	627.50

Note. Delta BIC = alternative BIC minus preferred-model BIC; positive values favor the preferred model. Global comparisons use all 12 items; pairwise comparisons use the eight items of the named pair. Source: P5_MODEL_COMPARISONS.csv; identical values appear in Table S8.

S2.2 Standardized item loadings

Standardized loadings ranged from .682 to .793 in Study 1 and from .668 to .777 in Study 2 (Table S2.3). All 24 loadings were positive, admissible, and above the conventional .60 threshold; the lowest values were AU3 in Study 1 (.682) and NA1/NA2 in Study 2 (.668).

Table S2.3. Standardized item loadings and residual variances from the focal three-factor CFAs.

Study	Factor	Item	Standardized loading	Standardized residual variance
Study 1	Temporal transportation	TT1	.753	.432
Study 1	Temporal transportation	TT2	.793	.371
Study 1	Temporal transportation	TT3	.720	.482
Study 1	Temporal transportation	TT4	.753	.433
Study 1	Nostalgic affect	NA1	.745	.445
Study 1	Nostalgic affect	NA2	.745	.446
Study 1	Nostalgic affect	NA3	.756	.429
Study 1	Nostalgic affect	NA4	.756	.428
Study 1	Visitor-perceived authenticity appraisal	AU1	.722	.479
Study 1	Visitor-perceived authenticity appraisal	AU2	.759	.424
Study 1	Visitor-perceived authenticity appraisal	AU3	.682	.535
Study 1	Visitor-perceived authenticity appraisal	AU4	.771	.406
Study 2	Temporal transportation	TT1	.706	.502
Study 2	Temporal transportation	TT2	.681	.536
Study 2	Temporal transportation	TT3	.702	.507
Study 2	Temporal transportation	TT4	.695	.518
Study 2	Nostalgic affect	NA1	.668	.554
Study 2	Nostalgic affect	NA2	.668	.554
Study 2	Nostalgic affect	NA3	.717	.486
Study 2	Nostalgic affect	NA4	.714	.491
Study 2	Visitor-perceived authenticity appraisal	AU1	.769	.409
Study 2	Visitor-perceived authenticity appraisal	AU2	.729	.469
Study 2	Visitor-perceived authenticity appraisal	AU3	.741	.451
Study 2	Visitor-perceived authenticity appraisal	AU4	.777	.397

Note. Source: P5_STANDARDIZED_LOADINGS.csv; identical values appear in Table S2 (Study 1) and Table S8 (both studies).

S2.3 Reliability and convergent validity

Alpha and composite reliability ranged from .823 to .841 in Study 1 and from .785 to .840 in Study 2 (Table S2.4). AVE exceeded .50 for all Study 1 constructs and for Study 2 authenticity appraisal. Study 2 AVE was .485 for temporal transportation and .479 for nostalgic affect, marginally below .50. These values are marginal when AVE is considered alone; no item was removed to force a threshold crossing, and the qualification is stated explicitly in the manuscript. We therefore regard the focal measurement evidence as broadly supportive but not perfect: reliability, loading magnitudes, overall fit, discriminant-validity statistics, and comparative-model evidence support the three-factor interpretation, while the Study 2 convergent-validity qualification remains explicit.

Table S2.4. Reliability, convergent validity, and loading ranges for the focal constructs.

Study	Construct	Items	M	SD	Alpha	CR	AVE	Loading range
Study 1	Temporal transportation	4	5.08	1.33	.841	.841	.571	.720–.793
Study 1	Nostalgic affect	4	5.23	1.30	.837	.838	.563	.745–.756
Study 1	Visitor-perceived authenticity appraisal	4	5.15	1.31	.823	.824	.539	.682–.771
Study 2	Temporal transportation	4	5.45	1.13	.790	.790	.485	.681–.706
Study 2	Nostalgic affect	4	5.60	1.11	.785	.786	.479	.668–.717
Study 2	Visitor-perceived authenticity appraisal	4	5.43	1.27	.840	.840	.568	.729–.777

Note. Alpha = Cronbach's alpha; CR = composite reliability; AVE = average variance extracted. Source: P5_RELIABILITY_VALIDITY.csv; identical values appear in Tables S1 (Study 1) and S6 (Study 2) and in manuscript Table 1.

Table S1 reports CFA-based average variance extracted (AVE) and composite reliability (CR) for the focused Study 1 models: Panel A covers the three focal constructs as operationalized in this paper, Panel B covers the Study 1 cue block (sensory staging, narrative coherence, participation architecture), and Panel C retains the remaining broader Study 1 construct summaries. Read together with Table S6 and Table S2.4, these summaries show a bounded within-project pattern: the focused Study 1 panels exceed 0.50, whereas Study 2 TT and NA retain acceptable α/CR with AVEs just below 0.50.

Note that in Table S1, Panel A reports CFA-based reliability summaries for the three focal constructs as operationalized in this paper, including the project-specific temporal-transportation measure used to capture past-oriented experiential presence in staged heritage settings. These values support the manuscript's within-project measurement argument but are not intended as stand-alone validation against all neighboring constructs. Panel B adds a focused Study 1 cue-block CFA for sensory staging, narrative coherence, and participation architecture because H2 depends on their separation. Panel C retains the remaining broader Study 1 construct summaries derived from the seven-factor EFA used elsewhere in the package, including the brief Study 1 perceived-sustainability-legitimacy scale as supplementary evidence outside the focal latent block.

S2.4 Factor correlations, HTMT, and within-project discriminant evidence

Maximum HTMT was .428 in Study 1 and .284 in Study 2, well below the .85 criterion for every pair (Table S2.5). Latent factor correlations were moderate in Study 1 (TT–NA = .386, TT–AU = .426, NA–AU = .279) and weaker in Study 2 (TT–NA = .028, TT–AU = .284, NA–AU = .199).

Table S2.5. Latent factor correlations, HTMT, and observed scale correlations for the focal construct pairs.

Study	Construct pair	Latent correlation	HTMT	Observed scale correlation
Study 1	TT-NA	.386	.387	.325
Study 1	TT-AU	.426	.428	.356
Study 1	NA-AU	.279	.279	.232
Study 2	TT-NA	.028	.053	.022
Study 2	TT-AU	.284	.284	.231
Study 2	NA-AU	.199	.205	.167

Note. HTMT = heterotrait-monotrait ratio of correlations. Sources: P5_HTMT.csv and measurement_cfa_factor_correlations.csv; identical values appear in Table S8.

To document the main-text construct-separation summary, we triangulate alpha/CR-AVE summaries, HTMT, comparisons of the three-factor CFA with one-factor and pairwise-collapsed alternatives, the TT2-exclusion sensitivity, and the robust/interval evidence in Table S22. Table S16 is a reduced pooled/unadjusted latent reparameterization, not a full robustness or invariance test. Together, these checks provide within-project calibration; they do not establish exhaustive validation against every neighboring construct.

Study 2 is especially informative because all TT, NA, and authenticity-appraisal items were answered in the same post-vignette sitting. Its main limitation is convergent variance: Tables S2.4 and S6 show acceptable α/CR but TT and NA AVEs of 0.485 and 0.479, and the experiment did not include concurrent neighboring constructs such as narrative transportation, general presence or immersion, or autobiographical mental time travel. At the same time, TT and NA were only weakly related in Study 2 (scale $r = 0.022$; latent $r = 0.028$; HTMT = 0.053), and the three-factor specification fit better than the collapsed alternatives in Tables S2.2 and S8. The shift from moderate TT–NA coupling in Study 1 to near-zero coupling in Study 2 likely also reflects the different elicitation modes—live post-experience responses versus brochure-based imagined judgments—so the TT2-exclusion analysis is interpreted as a targeted sensitivity to the most absorption-like item. The reduced latent models in Table S16 ask whether the positive TT–authenticity pattern remains under a leaner parameterization. Overall, Tables S2.1–S2.5, S6, S8, and

S16 provide cautious within-project discriminant evidence for the focal analyses while leaving broader scale-comparison questions open.

S2.5 Item-level descriptive statistics

For transparency, Tables S4 and S5 report item-level means, standard deviations, skewness, kurtosis, corrected item–total correlations, and missingness for Study 1 and Study 2, respectively. These diagnostics document distributional properties, potential ceiling effects, and the coherence of each item within its scale.

Table S6 presents descriptive summaries for the main Study 2 measures and diagnostic composites. Panel A reports TT, NA, and authenticity appraisal from the three-factor CFA, plus attendance intention as an exploratory nomological extension. The key convergent-evidence caveat is that TT and NA AVE values are 0.485 and 0.479 even though alpha and CR remain acceptable. These summaries are therefore interpreted with HTMT, preference for the three-factor structure over collapsed alternatives, the TT2-exclusion sensitivity, and Table S16's reduced latent reparameterization. Study 2 did not include concurrent neighboring constructs such as general presence or immersion, so this remains within-project calibration. Panel B reports manipulation-diagnostic composites only; these are uptake checks, not substantive latent measurement models.

S2.6 Numerical stability, independent implementation, and admissibility

Every primary model was estimated from 16 deterministic starting values. All optimizer calls converged to admissible solutions, and the preferred three-factor specification reached the same solution from all starts in both studies (Table S2.6). A second implementation used marker-variable identification and a Cholesky parameterization of the latent covariance matrix; it reproduced the same global solutions to numerical precision (maximum absolute chi-square difference 7.65×10^{-10}; maximum absolute loading difference 6.75×10^{-7}; maximum absolute factor-correlation difference 2.88×10^{-7}). Sample covariance matrices were well conditioned (minimum eigenvalue .877 in Study 1 and .866 in Study 2; condition numbers 12.68 and 8.77), model-implied covariance and latent covariance matrices were positive definite, the minimum standardized residual variance was .371 (Study 1) and .397 (Study 2), and no Heywood case occurred. All focal items used seven observed response categories with no missing values in the analytic data. Maximum absolute item skew was 1.081 and maximum absolute excess kurtosis was .602.

The planned covariance-based ML estimator was retained for the primary analysis. An authorized sensitivity subsequently applied Satorra-Bentler mean scaling with Huber-White sandwich parameter SEs and fixed-seed case-bootstrap AVE/HTMT intervals (Table S22). The scaled solutions were admissible in both studies; standardized loadings, CR, and AVE estimates were materially unchanged across implementations (maximum absolute difference = .00023). This sensitivity does not remove the mixed Study 2 convergent-evidence qualification.

Table S2.6. Multi-start convergence summary for the focal CFA models.

Study	Model	Starts	Successful	Starts at best tolerance	All admissible	Discrepancy range
Study 1	One-factor	16	16	16	Yes	1.74e-12
Study 1	Three-factor	16	16	16	Yes	7.74e-13
Study 1	TT+NA collapsed	16	16	16	Yes	1.72e-12
Study 1	TT+AU collapsed	16	16	16	Yes	1.44e-12
Study 1	NA+AU collapsed	16	16	16	Yes	3.40e-12
Study 2	One-factor	16	16	16	Yes	1.55e-12
Study 2	Three-factor	16	16	16	Yes	8.56e-13
Study 2	TT+NA collapsed	16	16	13	Yes	5.09e-02
Study 2	TT+AU collapsed	16	16	16	Yes	7.07e-13
Study 2	NA+AU collapsed	16	16	16	Yes	8.95e-13

Note. Discrepancy range = spread of the maximum-likelihood discrepancy across the 16 starts. Source: locked Phase 5 supplementary measurement module.

S2.7 Relationship to the substantive models

The CFAs above evaluate focal measurement structure. Primary hypothesis tests use unit-weighted scale means in HC3 OLS regressions (manuscript Tables 3 and 5; exact coefficients in Table S15). The analysis in S3.4 and Table S16 is a reduced pooled/unadjusted latent reparameterization, not a full robustness or invariance test. Measurement evidence precedes the observed-score hypothesis models.

S3. Robustness analyses

Section S3 assembles item-overlap, specification, leverage, and sample-composition checks behind the manuscript's main results. These analyses do not extend the paper's inferential reach. Table S16 is included as a reduced unadjusted reparameterization and is not treated as proof that the primary conclusions are model-independent. Baseline regressions use HC3 standard errors. Study 1 models also adjust for age, gender, driver/co-driver role, repeat attendance, log travel distance, and event fixed effects; the fixed effects absorb mean event differences but do not substitute for modeling within-event dependence. Table S7 also adds a parallel TT x legitimacy specificity check.

S3.1 Study 1: event composition, role heterogeneity, and influential cases

This section concentrates the main Study 1 robustness checks in one place. Table S7 places the SS1–SS3 no-overlap sensitivity and the Study 1 TT2-exclusion sensitivity alongside the S7_EventAnalyses composition sheet, leave-one-event-out ranges, and separate within-event re-estimations so readers can assess dependence on event composition, role mix, or one semantically proximate item. The SS1–SS3 no-overlap sensitivity preserves the sensory-staging → TT coefficient ($\beta = 0.34$, SE = 0.04, $p < 0.001$), and the TT–authenticity coefficient remains positive when TT is recomputed from TT1, TT3, and TT4 only ($\beta = 0.29$, SE = 0.04, $p < 0.001$). Across the four leave-one-event-out re-estimations, both the sensory-staging → TT coefficient and the TT → authenticity coefficient remained positive. The nostalgic affect × legitimacy term also remained positive, though less precise in the smaller event-specific subsets. Separate within-event re-estimations retained a positive TT pattern in every event.

S3.2 Study 2: specification and leverage sensitivity

Because Study 2 randomized brochure descriptions, we also re-estimated the condition models without demographic covariates; the substantive randomized pattern was unchanged. Table S7 places the direct Model C, the reduced and full adjusted Model D specifications, the interaction-inclusive adjusted Model D, the focused TT2-exclusion check, the specificity check with a parallel TT × sustainability-legitimacy term, and the AU1–AU3 authenticity sensitivity in one location. Across these specifications, the direct brochure interaction is confined to the simpler direct model and attenuates when AU4 is omitted or when the interaction is re-estimated alongside TT and NA ($\beta = 0.33$, $p = 0.037$ in the direct randomized model; $\beta = 0.28$, $p = 0.071$ in the AU1–AU3 direct sensitivity; $\beta = 0.25$, $p = 0.101$ in the interaction-inclusive adjusted model; $\beta = 0.20$, $p = 0.182$ in the AU1–AU3 interaction-inclusive sensitivity). By contrast, the TT coefficient remains positive across the adjusted and AU1–AU3 specifications.

S3.3 Diagnostic checks

Multicollinearity was low to moderate in the main models (maximum VIF = 1.46 for the Study 1 authenticity-appraisal model; 1.46 for the Study 1 authenticity-appraisal model with the added TT × legitimacy term in the focused specificity check; 3.02 for the Study 2 direct authenticity-appraisal condition model; 1.09 for the Study 2 reduced adjusted authenticity-appraisal specification; 2.03 for the Study 2 full adjusted authenticity-appraisal model; 2.07 for the Study 2 full adjusted authenticity-appraisal model with the added TT × legitimacy term in the focused specificity check; and 3.21 for the Study 2 interaction-inclusive adjusted authenticity-appraisal model). Tables S9–S10 provide a compact manipulation-targeting check for Study 2: the sensory manipulation strongly shifted perceived sensory richness but barely moved perceived sustainability legitimacy, whereas the higher/lower sustainability-legitimacy framing bundle strongly shifted perceived sustainability legitimacy but barely moved perceived sensory richness. This supports the intended distinction between the two brochure manipulations. Because the sustainability framing also varies transparency, seriousness, auditability, emissions-management credibility, and community-benefit signaling, the checks are uptake diagnostics for a broader framing bundle. Table S7 keeps the conservative no-overlap, TT2-exclusion, AU1–AU3, and moderation-specificity rows in one place; across these checks, the TT coefficient remains positive, whereas the Study 2 direct brochure interaction is less stable across specifications.

Specifically, Table S7 presents robustness checks for main coefficients (Studies 1 and 2). Baseline models use HC3 robust standard errors. Study 1 baseline models include participant covariates and event fixed effects, which absorb mean differences across the four events but do not provide a strong event-level test. The companion by-event sheet in Table S7 reports event-by-role composition and separate single-event re-estimations for the self-selected analytic subset. Study 1 cue variables are respondent perceptions of event design. The SS1–SS3 no-overlap sensitivity recomputes sensory staging from SS1–SS3 only, excluding SS4; the TT2-exclusion sensitivity recomputes TT from TT1, TT3, and TT4 only; and the AU1–AU3 sensitivity omits AU4, the credibility-adjacent authenticity item. Focused specificity rows add a parallel TT × legitimacy term. Because perceived sustainability legitimacy was measured after authenticity appraisal and the exploratory-extension blocks, the Study 1 moderation rows remain later-measured conditional associations. For Study 2, Model C and the Model D variants are shown together. All displayed models use OLS with HC3 SEs. Models A-C include the two assigned 0/1 indicators, their product, z-

standardized age, and Female/Other indicators (male reference). Model D (reduced) adds z-standardized TT to assigned main effects and controls; Model D (full) adds z-standardized NA and NA x sustainability, and the interaction-inclusive form also restores sensory x sustainability. Outcomes and continuous predictors are standardized; binary indicators remain 0/1. Brochure terms are randomized contrasts; links among post-treatment states remain adjusted within-session associations. Table S7 also reports direct exploratory same-model Wald contrasts of NA x legitimacy versus TT x legitimacy in the same adjusted specificity models (Study 1: difference = .209, SE = .071, 95% CI [.070, .348], p = .003; Study 2: difference = .286, SE = .104, 95% CI [.082, .490], p = .006). These non-preregistered contrasts are specificity checks, not confirmatory pathway tests.

Table S13 compares focal Study 2 coefficients in the final analytic sample (N = 640) with the completed sample before quality-control exclusions (N = 660). Entries are OLS estimates with HC3 SEs. Outcomes and continuous predictors are z-standardized; binary indicators remain 0/1. Models A-C include assigned indicators, their product, age, and Female/Other controls; Model D rows add the stated within-session predictors.

Panel B retains exploratory association-chain summaries based on 5,000 resamples and is not a mediation analysis. Because only the brochure descriptions were randomized and post-vignette measures were collected once, these summaries do not identify indirect causal effects. The higher/lower sustainability contrast remains a bundled framing manipulation rather than an isolated single-cue treatment.

Table S14 presents Study 2 demographic descriptors by experimental cell (final analytic sample). Age is shown as mean (SD); categorical descriptors are n (%). 'Other / prefer not to say' combines the Non-binary/Other and Prefer not categories. Region uses the non-sensitive UK country categories retained in the completed-sample file.

Table S15 presents exact coefficient reporting for main models and summary indices. Panels A–B report the exact term-level coefficients for the manuscript's adjusted observed-variable regression models, estimated on unit-weighted composites, with coefficient estimates, HC3 robust SEs, 95% confidence intervals, exact p values, sample sizes, and model R^2. Here TT refers to the paper's project-specific temporal-transportation operationalization. In Study 1, these rows summarize adjusted field associations among attendee perceptions, later respondent judgments, and reported states/appraisals. In Study 2, the main-text sequence comprises Model A, Model B, Model C, and the parallel Model D authenticity-appraisal specifications (reduced, full, and interaction-inclusive); the attendance-intention model is retained as an exploratory nomological extension, and TT2-exclusion rows are retained so the construct-distinction sensitivity remains visible. The Study 2 sustainability term refers to the broader higher/lower sustainability-legitimacy framing bundle. Panel C reports the Study 1 bootstrapped exploratory association-chain summaries through place attachment and advocacy, with the corresponding summary index. Panel D reports the Study 2 bootstrapped association-chain summaries and index for the exploratory attendance-intention extension. Both panels use 5,000 resamples and do not identify sequential or causal mediation. Panel E reports conditional TT-versus-NA comparisons for the Study 1 nostalgic-affect × legitimacy interaction and the aligned Study 2 higher/lower-legitimacy comparisons, with TT2-exclusion and AU1–AU3 sensitivity rows shown separately. Panel F reports AU1–AU3 sensitivity coefficients after omitting AU4, the credibility-adjacent authenticity item. The companion SS1–SS3 no-overlap sensitivity appears in Table S7.

S3.4 Reduced latent-variable reparameterization

Table S16 reproduces reduced latent regressions for the TT-NA-authenticity-appraisal block. Authenticity appraisal is an endogenous latent factor regressed on latent TT and NA for Study 1, pooled Study 2, and the two Study 2 framing subsets. The same indicators used in the corresponding CFA are retained.

These pooled and split rows are unadjusted descriptive reparameterizations. They omit the full covariate set, the direct brochure terms, and the interaction structure used in the primary observed-variable models. The framing-split rows are not a formal measurement-invariance analysis and do not test whether coefficients differ between frames. They therefore cannot establish that every conclusion is model-independent or that measurement parameters are invariant across groups.

The positive latent TT coefficients provide a limited within-project sensitivity for the direction of that association. The numerical table is retained only as a reduced reparameterization and should be interpreted with the primary HC3 models, the TT2-exclusion sensitivity, and Table S22. No causal interpretation is attached to the post-treatment latent associations in Study 2.

Table S3.1. Reduced latent-regression reparameterization (values reproduced from Table S16).

Sample	N	chi-square (df)	CFI	TLI	RMSEA	SRMR	Latent r(TT, NA)	Std. β TT	Std. β NA	R^2 authenticity appraisal
Study 1: full sample	520	49.36 (51)	1.000	1.000	.000	.020	.386	.374	.135	.197
Study 2: pooled sample	640	53.91 (51)	.999	.998	.009	.028	.028	.279	.191	.117
Study 2: lower sustainability-legitimacy framing	320	51.10 (51)	1.000	1.000	.003	.038	.015	.300	.067	.095
Study 2: higher sustainability-legitimacy framing	320	56.06 (51)	.996	.995	.018	.036	.035	.223	.320	.157

Note. Authenticity appraisal is modeled as an endogenous latent factor regressed on latent temporal transportation and latent nostalgic affect; Study 2 is shown pooled and split by sustainability-legitimacy description. Fit reporting uses the effective identified 51 degrees of freedom. Source: measurement_latent_core_robustness.csv (Table S16).

S3.5 Integrative summary tables

Section S3.5 consolidates the bounded synthesis. Table S17 separates cross-context recurrence, conditional/context-bounded patterns, and context-specific or exploratory extensions. Table S18 gathers the direct randomized Study 2 authenticity pattern, Model C coefficients, and the AU1-AU3 sensitivity cross-reference; Tables S19-S20 retain the exploratory extensions.

Table S17 presents the bounded cross-context synthesis. Rows separate cross-context recurrence, conditional/context-bounded patterns, and context-specific or exploratory extensions. Study 1 entries are adjusted field associations. In Study 2, assigned brochure terms are randomized contrasts; rows involving TT, NA, or authenticity appraisal are adjusted within-session associations conditional on treatment. H5 remains partial/conditional and H6 qualified.

Table S18 integrates Study 2 cell means, key randomized brochure-condition contrasts, and the covariate-adjusted Model C authenticity-appraisal condition model. Model C includes the two assigned brochure indicators, their product, z-standardized age, and Female/Other indicators (male reference); outcomes are standardized and binary indicators remain 0/1.

The higher/lower sustainability-legitimacy manipulation is a bundled description and does not isolate individual message components. The interaction is reliable in Model C (beta = .33, p = .037), attenuates in the AU1-AU3 sensitivity (beta = .28, p = .071), and is imprecise in the interaction-inclusive adjusted model (beta = .25, p = .101).

Table S19 presents the Study 1 exploratory nomological extensions: the memorability, place-attachment, and advocacy models together with the corresponding association-chain summaries. These displays sit outside the core theory tests; because the variables were measured in the same session, they are reported as exploratory nomological extensions.

Table S20 presents the Study 2 exploratory nomological extension: the attendance-intention model and the corresponding association-chain summaries. Because these estimates combine randomized brochure-condition contrasts with adjusted within-session associations conditional on treatment from one post-vignette session, they are reported as exploratory nomological extensions.

S4. Study 2 stimuli

Study 2 used a generic, non-branded base brochure description combined with two randomized, crossed text-framing manipulations: described sensory richness (low vs. high) and a higher/lower sustainability-legitimacy framing bundle. Respondents evaluated described event materials, not a live event. At the text level, the sensory contrast bundled vividness and multisensory imagery, whereas the higher sustainability-legitimacy framing bundled reporting transparency, independent audit, emissions-management, heritage-conservation, and community-benefit cues, and the lower framing contained skeptical cues about unclear reporting and symbolic practice. The manipulations were well targeted at the composite level (Tables S9–S10) but were not designed to isolate one pure cue dimension or a single legitimacy cue. The lower framing was not an extreme denunciation, so the experiment identifies relative shifts between bundled brochure descriptions. The English-language wording used in the survey appears below. In the survey software, the base vignette and the two manipulation paragraphs were combined according to random assignment.

S4.1 Base vignette (shared across conditions)

Imagine a two-day historic road rally held in a European destination. Participants drive classic vehicles along a scenic route with several public checkpoints. Spectators can watch arrivals, see the cars up close, and enjoy local food and activities at the start/finish areas.

S4.2 Described sensory richness manipulation

Low sensory condition (general descriptive wording with limited vividness/detail and multisensory imagery): The event is described in general terms. Cars arrive at checkpoints and continue along the route. People can watch, take photos, and see vehicles parked in the town square.

High sensory condition (greater vividness/detail and multisensory imagery): The description emphasizes multisensory detail. As cars arrive, you hear the distinctive rasp and cadence of engines, smell fuel and warm oil, and feel brief vibration as vehicles idle nearby. Chrome, leather, and period design details stand out, and the crowd ambience (voices, music, and announcements) reinforces the impression of another era.

S4.3 Lower versus higher sustainability-legitimacy framing bundle

Lower sustainability-legitimacy framing bundle (skeptical cues about unclear reporting, limited evidence of serious follow-through, weak auditability, and largely symbolic emissions/community-benefit practice): The event website presents the rally positively and mentions sustainability, but provides few details. There is no clear reporting of emissions or community impacts, and the initiatives appear limited and mostly symbolic. The wording therefore signals unclear reporting and symbolic practice, and stops short of misconduct or falsification.

Higher sustainability-legitimacy framing bundle (reporting transparency, independent audit, emissions-management, heritage-conservation, and community-benefit cues): The event is described positively and as implementing credible sustainability practices, including transparent emissions accounting verified by an independent auditor, initiatives to reduce idling and logistics impacts, and a clearly communicated contribution to local heritage conservation and community benefits. The wording is designed to strengthen perceived legitimacy-related framing within the vignette; it does not independently verify real-world sustainability performance.

S5. Additional measurement summaries and analysis files

Tables S21 and S22 provide additional reliability and measurement-sensitivity summaries.

Table S21 reports verified Study 1 secondary-scale reliability and descriptive summaries: personal nostalgia proneness (alpha = .823, M = 3.944, SD = 1.436), historical nostalgia proneness (alpha = .838, M = 3.918, SD = 1.449), and memorability (alpha = .815, M = 5.229, SD = 1.342). Values were reproduced from the analytic data and code-defined item mappings.

Table S22 reports Satorra-Bentler mean-scaled ML fit, Huber-White sandwich loading SEs, fixed-seed 90% case-bootstrap intervals for AVE, and fixed-seed 95% case-bootstrap intervals for HTMT. AVE seeds were 4311859 (Study 1) and 4312859 (Study 2); HTMT seeds were 4311959 and 4312959. The Study 2 TT and NA AVE intervals cross .50, so this sensitivity retains the manuscript's mixed-convergent-evidence qualification rather than converting it into an unqualified validity claim.

All Tables S1–S22 are supplied in behavsci-4311859-supplementary-tables.xlsx. Its Contents worksheet lists all 47 numbered-table worksheets and the PDF_Tables worksheet, which reproduces the seven appendix-specific displays. The machine-readable analysis outputs and fixed-seed script supporting Table S22 are included in Authorized_Measurement_Sensitivity_Analysis.zip.
